# Wearable Artificial Intelligence for Sleep Disorders: Scoping Review

**DOI:** 10.2196/65272

**Published:** 2025-05-06

**Authors:** Sarah Aziz, Amal A M Ali, Hania Aslam, Alaa A Abd-alrazaq, Rawan AlSaad, Mohannad Alajlani, Reham Ahmad, Laila Khalil, Arfan Ahmed, Javaid Sheikh

**Affiliations:** 1 AI Center for Precision Health Weill Cornell Medicine-Qatar Doha Qatar; 2 College of Science and Engineering Hamad Bin Khalifa University Doha Qatar; 3 Social and Economic Survey Research Institute Qatar University Doha Qatar; 4 Institute of Digital Healthcare University of Warwick Warwick United Kingdom; 5 Weill Cornell Medicine-Qatar Doha Qatar

**Keywords:** sleep disorders, wearable devices, artificial intelligence, machine learning, scoping review

## Abstract

**Background:**

Worldwide, 30%-45% of adults have sleep disorders, which are linked to major health issues such as diabetes and cardiovascular disease. Long-term monitoring with traditional in-lab testing is impractical due to high costs. Wearable artificial intelligence (AI)–powered solutions offer accessible, scalable, and continuous monitoring, improving the identification and treatment of sleep problems.

**Objective:**

This scoping review aims to provide an overview of AI-powered wearable devices used for sleep disorders, focusing on study characteristics, wearable technology features, and AI methodologies for detection and analysis.

**Methods:**

Seven electronic databases (MEDLINE, PsycINFO, Embase, IEEE Xplore, ACM Digital Library, Google Scholar, and Scopus) were searched for peer-reviewed literature published before March 2024. Keywords were selected based on 3 domains: sleep disorders, AI, and wearable devices. The primary selection criterion was the inclusion of studies that utilized AI algorithms to detect or predict various sleep disorders using data from wearable devices. Study selection was conducted in 2 steps: first, by reviewing titles and abstracts, followed by full-text screening. Two reviewers independently conducted study selection and data extraction, resolving discrepancies by consensus. The extracted data were synthesized using a narrative approach.

**Results:**

The initial search yielded 615 articles, of which 46 met the eligibility criteria and were included in the final analysis. The majority of studies focused on sleep apnea. Wearable AI was widely deployed for diagnosing and screening disorders; however, none of the studies used it for treatment. Commercial devices were the most commonly used type of wearable technology, appearing in 30 out of 46 (65%) studies. Among these, various brands were utilized rather than a single large, well-known brand; 19 (41%) studies used wrist-worn devices. Respiratory data were used by 25 of 46 (54%) studies as the primary data for model development, followed by heart rate (22/46, 48%) and body movement (17/46, 37%). The most popular algorithm was the convolutional neural network, adopted by 17 of 46 (37%) studies, followed by random forest (14/46, 30%) and support vector machines (12/46, 26%).

**Conclusions:**

Wearable AI technology offers promising solutions for sleep disorders. These devices can be used for screening and diagnosis; however, research on wearable technology for sleep disorders other than sleep apnea remains limited. To statistically synthesize performance and efficacy results, more reviews are needed. Technology companies should prioritize advancements such as deep learning algorithms and invest in wearable AI for treating sleep disorders, given its potential. Further research is necessary to validate machine learning techniques using clinical data from wearable devices and to develop useful analytics for data collection, monitoring, prediction, classification, and recommendation in the context of sleep disorders.

## Introduction

### Background

Sleep is a fundamental biological process essential for maintaining overall health and well-being. It is a dynamic state in which the brain processes daily experiences, promotes synaptic plasticity, and supports physical functions. During sleep, the brain and body engage in recovery, repair, and preparation for the next day [1]. Sufficient sleep is crucial for mood stability, cognitive function, and overall health. Both sleep quantity and quality are vital for optimal functioning of the body and mind [2]. The National Sleep Foundation defines optimal sleep quantity for adults as 7-9 hours per night [3], while sleep quality is characterized by factors such as minimal interruptions, appropriate sleep onset latency (typically under 30 minutes), and a significant proportion of restorative sleep stages (eg, deep sleep, rapid eye movement sleep). According to the Philips Global Sleep Survey [4], 62% of people worldwide report not getting the quality of sleep they desire, and 44% have experienced worsening sleep over the past 5 years, a problem that may be attributed to various sleep disorders. The International Classification of Sleep Disorders categorizes sleep disorders into insomnia, sleep-disordered breathing, central hypersomnolence disorders, circadian rhythm sleep-wake disorders, parasomnias, and sleep-related movement disorders [5].

Existing research has shown that sleep disorders significantly impact both physical and mental health. They can manifest as insufficient sleep, excessive sleep, or abnormal movements during sleep. Several studies have found that sleep disorders are associated with an increased risk of cardiovascular disease, diabetes, and cancer [6-8]. Additionally, they are linked to mental health issues such as depression, anxiety, and suicidal behavior [9-11]. Beyond individual health, sleep disorders also have broader societal consequences, including an increased risk of road accidents [12]. To mitigate the negative health and social impacts of sleep disorders, early detection, monitoring, and treatment are essential.

Various methods and devices have been used to monitor and diagnose sleep disorders, including polysomnography (PSG), home sleep testing (HST), and actigraphy. PSG is the gold standard for diagnosing sleep disorders, as it accurately assesses sleep phases and identifies potential conditions. However, despite its advantages, PSG has some limitations. It is costly and time-consuming, requires individuals to spend the night in a sleep laboratory, and depends on expert monitoring and scoring. By contrast, HST and actigraphy are less costly, allow data collection over multiple days, and can be used in nonlaboratory settings compared with PSG [13,14]. However, HST has limitations, including underestimating results and providing limited evaluations of certain sleep disorders [15,16]. These limitations can be addressed by wearable artificial intelligence (AI) technology.

As wearable AI devices become increasingly popular, they have revolutionized the health care industry by enabling real-time monitoring and diagnostic capabilities [17]. This technology integrates AI into wearable devices (WDs), allowing them to perform tasks such as data processing, inference, and decision-making directly on the device [18]. According to the IEC (International Electrotechnical Commission) Standardization Group 10, wearable smart devices are categorized into 4 groups based on their proximity to, placement on, or implantation within an organism, such as the human body (as cited in [19]). Near-body wearables, such as radar-based monitoring systems, contactless sleep-tracking devices, and mobile sleep apps, operate close to the body but do not require direct skin contact. On-body wearables, including smartwatches, fitness trackers, smart glasses, electrocardiogram electrodes, electromyography sensors, and electrodermal activity monitors, are worn directly on the body and maintain continuous skin contact. In-body wearables, such as implantable smart patches and pacemakers, are implanted into the body. Electronic textiles integrate fabric-based electronics, including smart clothing designed to monitor physiological parameters.

### Research Problem and Aim

Several studies have been published on the use of WDs combined with AI to detect or monitor sleep disorders. While multiple reviews have summarized previous studies, certain limitations exist. Some reviews focused solely on the features of AI models without discussing their integration with WDs [20-22]. Others examined only a specific type of sleep disorder [23-25]. Additionally, several reviews used search queries that omitted important terms [22,26]. Numerous reviews did not include searches in popular databases such as MEDLINE, PsycINFO, and Embase [20-22]. Some reviews focused on specific types of data, such as clinical data or consumer data from sleep technology devices used outside clinical settings [20,26]. Several reviews were narrative in nature, indicating that they did not follow systematic approaches [22,26]. Therefore, this review aims to provide an overview of AI-powered WDs used for sleep disorders by analyzing key aspects across 3 dimensions. First, it examines the study characteristics, including design, population, and geographical trends, to highlight research patterns. Second, it explores the technological features of WDs, such as sensor types and biosignals collected, emphasizing their role in sleep monitoring. Third, it investigates the AI methodologies employed, their applications, and validation approaches, showcasing advancements in AI-driven sleep disorder detection.

## Methods

### Study Design

To ensure a thorough and systematic approach, this scoping review adhered to the PRISMA-ScR (Preferred Reporting Items for Systematic Reviews and Meta-Analyses extension for Scoping Reviews). A detailed account of adherence to PRISMA-ScR guidelines is provided in Multimedia Appendix 1, outlining the structured process we followed.

### Search Strategy

A comprehensive search was conducted across several electronic databases, including MEDLINE, PsycINFO, Embase, IEEE Xplore, ACM Digital Library, Scopus, and Google Scholar. An automatic alert was set to run the search query biweekly. The bibliographic data collection period spanned from December 7, 2023, to March 6, 2024. As a result of the overwhelming volume of results from Google Scholar and its ability to prioritize relevant search results, this review deliberately focused only on the first 100 results. To identify additional relevant sources, we performed backward and forward reference list checking. This process involved analyzing the reference lists of included articles and examining studies that cited them using Google Scholar’s “Cited by” feature.

The search queries combined terms related to sleep disorders (eg, sleep disorder*s, sleep disturbance, and sleep apnea) with terms related to AI (eg, AI, machine learning, and deep learning) and WDs (eg, wearable, smartwatch, and smart band). In collaboration with digital health experts and after reviewing relevant literature, the final search query was meticulously crafted. Boolean operators “OR” and “AND” were used to combine terms within the same category and across different categories, respectively. The language filter was set to English only. Duplicates were identified and removed using EndNote X9 (Clarivate Plc). Full details of the search terms used for each electronic database are provided in Multimedia Appendix 2.

### Study Eligibility Criteria

This review encompassed studies that utilized AI algorithms for any purpose related to sleep disorders using data from WDs. Research articles were deemed suitable for inclusion if they primarily focused on individuals diagnosed with or suspected of having any type of sleep disorder, without restrictions based on age, gender, or ethnicity. Studies that focused solely on AI applications for detecting sleep quality or sleep staging—without directly addressing sleep disorders—or those forecasting intervention outcomes for sleep disorders were excluded.

This review included studies that gathered data using noninvasive, on-body WDs. Research papers that exclusively relied on non-WDs, handheld devices (eg, mobile phones), near-body or in-body WDs, WDs physically connected to non-WDs, or wearables requiring expert oversight—such as those necessitating precise electrode placement—were excluded. Studies on animals or patients with other primary health conditions were also eliminated. Additionally, only peer-reviewed journal articles, conference papers, and dissertations were considered, with no restrictions on study setting, study design, reference standard (ie, ground truth), year of publication, or country of study. However, papers not published in English or classified as editorials, preprints, reviews, protocols, posters, conference abstracts, or research highlights were excluded from consideration.

### Study Selection Process

The study selection process in this review comprised 2 key steps. First, all retrieved articles underwent a preliminary screening based on their titles and abstracts by 2 reviewers. This step was essential for determining whether the articles met the inclusion criteria without requiring a full-text review. It aimed to exclude studies that clearly did not meet the criteria, such as those unrelated to WDs or focusing on other aspects of sleep technology.

Articles that passed the initial screening were then subjected to a detailed full-text review. The same 2 reviewers independently conducted this assessment, thoroughly evaluating each study against the inclusion and exclusion criteria to confirm its relevance to the research questions. Studies that lacked sufficient data on AI algorithm performance, used nonwearable technology, or fell outside the scope of peer-reviewed literature were excluded. Any discrepancies between reviewers were resolved through discussion until a consensus was reached. If disagreement persisted, a third reviewer was consulted to make the final decision.

### Data Extraction Process

Two reviewers independently extracted data from the included studies using Microsoft Excel. The extracted information included study metadata, WD features, and AI algorithm characteristics. The data extraction form used in this review is provided in Multimedia Appendix 3. Any differences in data interpretation or extraction between reviewers were resolved through discussion until a consensus was reached.

### Data Synthesis

We used a narrative approach to synthesize the extracted data, which were then aggregated using text, tables, and figures. Specifically, we first presented the search results, followed by an overview of the studies’ general characteristics, and finally, a detailed description of the features of WDs and AI technologies. We examined the technical characteristics of WDs, including key measurements, sensing approaches, and sensor properties, as well as general attributes such as device type, placement, and status. The AI aspects were analyzed based on the models used, evaluation criteria, and their applications.

## Results

### Search Findings

**Figure 1:**
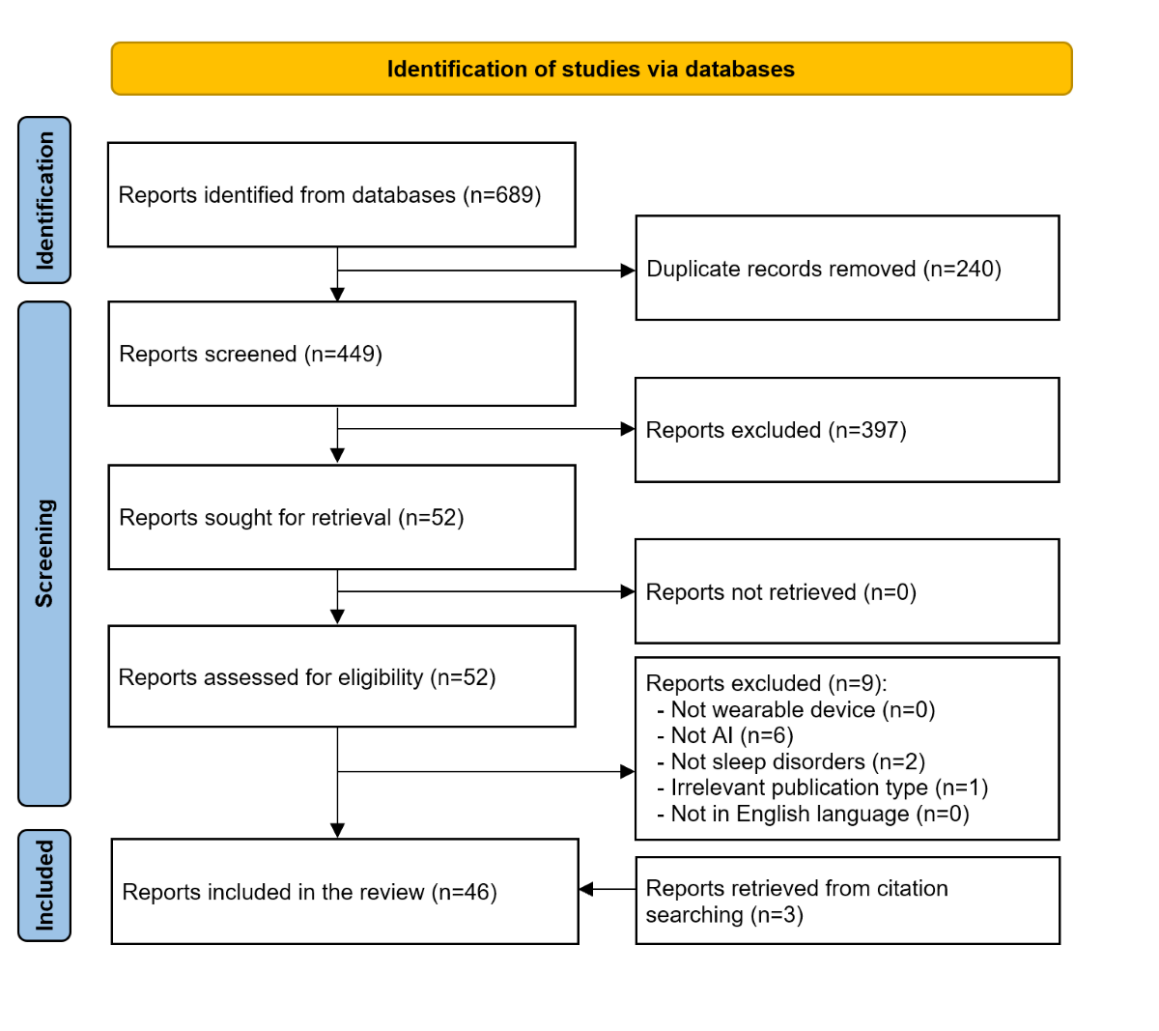
Flowchart of the study selection process. AI: artificial intelligence.

Figure 1 illustrates the study selection process, as per PRISMA-ScR guidelines (Multimedia Appendix 1). The initial database search yielded 689 citations. After identifying and removing 240 duplicates using EndNote X9, 449 unique studies remained. Screening the titles and abstracts led to the exclusion of 397 studies. The full texts of the remaining 52 studies were retrieved and assessed, resulting in the exclusion of 9 studies. The primary reasons for exclusion were the lack of studies focusing on sleep disorders (n=2), the absence of AI algorithms (n=6), and inappropriate publication type (n=1). Additionally, 3 relevant studies were identified through reference list screening. Ultimately, 46 studies were included in this review.

### Characteristics of Included Studies

As shown in Table 1, the number of studies fluctuated over time, with the highest counts recorded in 2023 and 2020 (11/46, 24%). The included studies were conducted across 17 different countries, with the United States contributing the most (10/46, 22%). The majority of the research was published as journal articles (36/46, 78%). The average number of participants per study was 218.4 (SD 597.2), ranging from 4 to 3414. Among the 29 studies that reported participant ages, the age range was 12-68 years, with an average of 45.8 (SD 12.4) years. The proportion of female participants across 30 studies averaged 39.2%, ranging from 12% to 65%. The majority of studies (42/46, 91%) focused on sleep apnea. Multimedia Appendix 4 provides the characteristics of each included study.

**Table 1:** Characteristics of the included studies (N=46).

Features			Studies		References
**Year of publication, n (%)**					
	2023	11 (24)		[27-37]	
	2022	10 (22)		[38-47]	
	2021	6 (13)		[48-53]	
	2020	11 (24)		[54-64]	
	2019	2 (4)		[65,66]	
	2018	3 (7)		[67-69]	
	Others^a^	3 (7)		[70-72]	
**Country of publication, n (%)**					
	United States	10 (22)		[30,33,40,48,51,58,69-72]	
	China	9 (20)		[34-37,39,41,44,45,50]	
	South Korea	5 (11)		[28,47,55,64,66]	
	Ukraine	3 (7)		[49,60,61]	
	Australia	2 (4)		[42,53]	
	Canada	2 (4)		[63,65]	
	Italy	2 (4)		[32,38]	
	Norway	2 (4)		[29,52]	
	Taiwan	2 (4)		[54,62]	
	The Netherlands	2 (4)		[31,59]	
	Others^a^	7 (15)		[27,43,55-57,67,68]	
**Publication type, n (%)**					
	Journal article	36 (78)		[27-36,38-48,50-59,62,63,66,69,71]	
	Conference paper	10 (22)		[37,49,60,61,64,65,67,68,70,72]	
**Number of participants**					
	Mean (SD)	218.4 (597.2)		[27-29,31-72]	
	Range	4-3414		N/A^b^	
	Not reported, n (%)	1 (2)		[30]	
**Age**					
	Mean (SD)	45.8 (12.4)		[28,29,31-33,35,36,38,40,42,43,45-49,51,52,54,55,57,59-63,65,66,69,70]	
	Range	12-68		N/A	
	Not reported, n (%)	17 (37)		[27,30,34,37,39,41,44,50,53,55,56,58,64,67,68,71,72]	
**Female (%)**					
	Mean (SD)	39.2 (14.2)		[28,29,32-36,38,40-43,45,46,48,51-54,57-60,62,63,65,66,69,70,72]	
	Range	12-65		N/A	
	Not reported, n (%)	16 (35)		[27,30,31,37,39,44,47,49,50,55,56,61,64,67,68,71]	
**Target disease, n (%)**					
	Sleep apnea	39 (84.7)		[29,30,33-41,43-52,54-65,67-72]	
	Insomnia	4 (9)		[28,42,53,66]	
	Rapid eye movement sleep behavior disorder	1 (2)		[31]	
	Sleep stroke	1 (2)		[27]	
	Sleep disorder^c^	1 (2)		[32]	

^a^Other includes the total number of studies where a feature was added as one.

^b^N/A: not applicable.

^c^Not any specific disorder, but in general, all characteristics considered for any specific disorder, such as breathing events, apnea, irregular breathing, snoring, and obstructive sleep apnea.

### Technical Specifications of Wearable Devices

**Figure 2:**
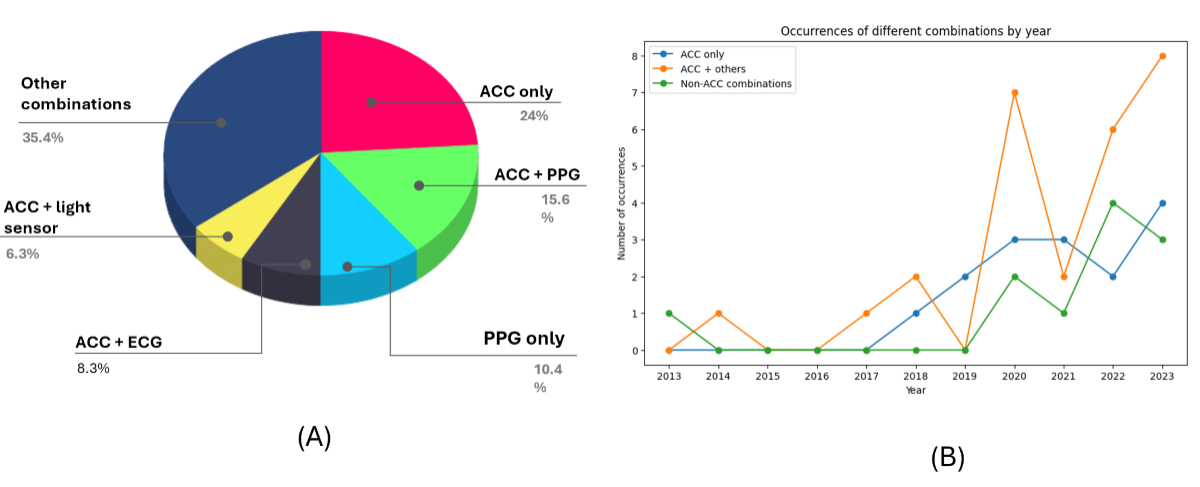
Sensor types used for sleep analysis by devices. ACC: accelerometer; ECG: electrocardiogram; PPG: photoplethysmography.

Commercial WDs constituted the majority of the included studies (30/46, 65%; Table 2). The most frequently mentioned WDs were Actiwatch, Belun Ring, and Fitbit (3/46, 7%), with smart bands being the most common type (12/46, 26%). WDs were placed on various body parts, with the wrist (19/46, 41%), chest (15/46, 33%), and abdomen (8/46, 17%) being the most common locations. Most of these devices collected activity and sleep measures (10/46, 22%), along with other biosignals. As illustrated in Figure 2A, the most common sensors identified in these WDs were accelerometers (34/46, 74%) and photoplethysmography sensors (14/46, 30%). Figure 2B highlights a clear trend of accelerometer sensor adoption over the years, often in combination with other sensors. Most of these devices (44/46, 96%) employed an opportunistic approach to data collection, autonomously sensing and recording data without requiring users to manually input information or activate processes. The technical specifications of the WDs in each included study are detailed in Multimedia Appendices 5 and 6.

**Table 2:** Technical specifications of wearable devices.

Feature				Studies, n (%)		References
**Status of WD^a^**						
		Commercial	30 (65)		[28,29,31-33,37-40,42-44,46-53,56-58,61,62,64,66,67,70,72]	
		Noncommercial	16 (35)		[27,30,34-36,41,45,54,55,59,60,63,65,68,69,71]	
**Name of WD**						
		Actiwatch	3 (7)		[42,53,67]	
		Belun Ring	3 (7)		[33,51,62]	
		Fitbit	3 (7)		[28,38,66]	
		ADXL345	2 (4)		[55,69]	
		Alice 5 PSG	2 (4)		[54,69]	
		Patch	2 (4)		[63,65]	
		Samsung Galaxy	2 (4)		[49,61]	
		T-REX TR100A	2 (4)		[46,47]	
		Not reported	13 (28)		[27,30,34,36,39,41,44,45,50,59,60,68,71]	
		Others	15 (33)		[29,31,32,35,37,40,43,48,52,56-58,64,70,72]	
**Type of WD**						
		Smart band	12 (26)		[27,31,38-40,42,44,50,53,57,59,66]	
		Smartwatch	8 (17)		[28,37,48,49,61,64,67,72]	
		Electrodes	7 (15)		[30,41,46,47,54,56,70]	
		Smart ring	4 (9)		[33,34,51,62]	
		Sensor	3 (7)		[35,36,69]	
		Smart belt	2 (4)		[29,55]	
		Not reported	1 (2)		[45]	
		Others	2 (4)		[52,71]	
**Placement**						
		Wrist	19 (41)		[27,28,31,37-39,42,44,48-50,52,53,59,61,64,66,67,72]	
		Chest	15 (33)		[29,32,40,41,43,45,52,54,56-58,60,68,70,71]	
		Abdomen	8 (17)		[29,46,47,52,54-56,58]	
		Finger	6 (13)		[29,33,34,51,52,62]	
		Nose	3 (7)		[29,36,52]	
		Neck	2 (4)		[63,65]	
		Not reported	1 (2)		[69]	
		Others	2 (4)		[30,35]	
**Measured biosignals**						
		Activity measures	34 (74)		[27-29,31-33,37,38,40-43,48,49,51-55,57-70,72]	
		Sleep measures	34 (74)		[27-29,31-33,37,38,40-43,48,49,51-55,57-70,72]	
		Cardiovascular measures	23 (50)		[33,34,37-39,41,44-47,49-51,54,57,59,61,62,64,68,70-72]	
		Oxygenation measures	16 (35)		[33-35,37-39,44,49-51,59,61,62,64,69,72]	
		Light exposure	4 (9)		[31,42,49,53]	
		Motion measures	2 (4)		[27,58]	
		Respiratory data	2 (4)		[36,56]	
		Others	3 (7)		[27,30,32]	
**Sensors**						
	Accelerometer			34 (74)		[27-29,31-33,37,38,40-43,48,49,51-55,57-70,72]
	Photoplethysmography			14 (30)		[33,34,37-39,44,49-51,59,61,62,64,72]
	Light sensor			5 (11)		[31,35,42,49,53]
	Electrocardiogram			9 (20)		[41,45,47,54,55,57,68,70,71]
	Gyroscope			2 (4)		N/A^b^
	Others			9 (20)		[27,30,32,36,56,58,62,69,71]
**Sensing approach**						
	Opportunistic			44 (96)		[27-50,52-67,69-72]
	Participatory			2 (4)		[51,53]
	Not reported			1 (2)		[68]

^a^WD: wearable device.

^b^N/A: not applicable.

### AI Model Charateristics

**Figure 3:**
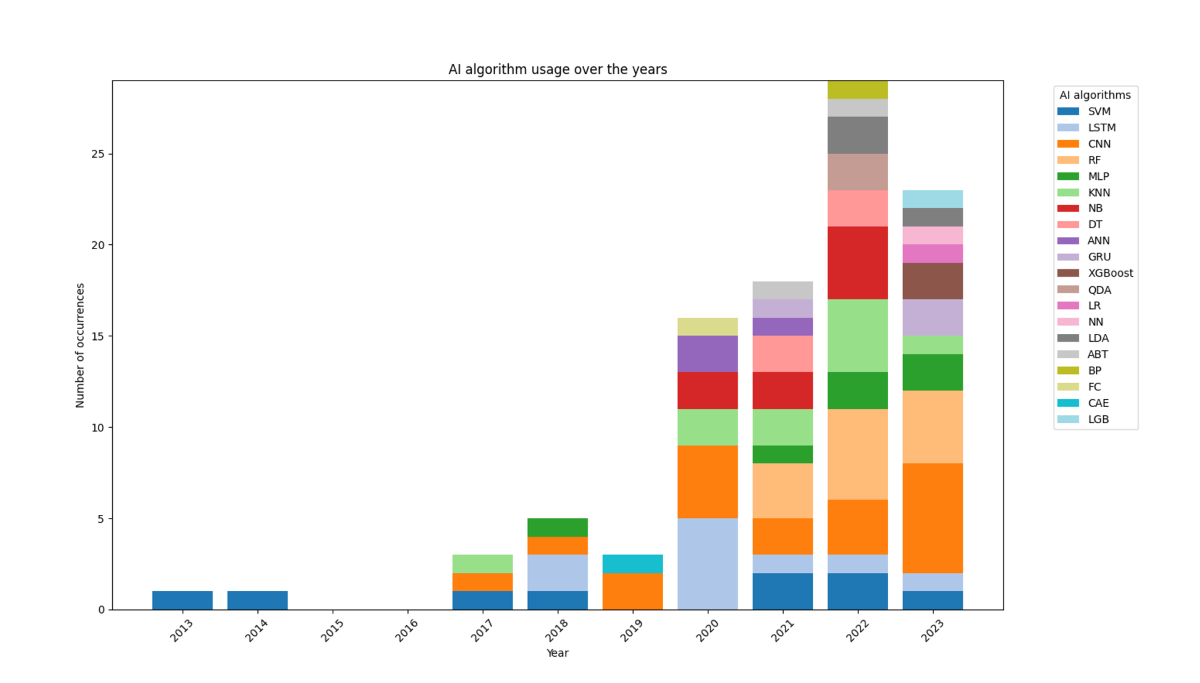
Artificial intelligence (AI) algorithm usage over the years. ABT: Adaptive Boosting; ANN: artificial neural network; BP: backpropagation; CAE: convolutional autoencoder; CNN: convolutional neural network; DT: decision tree; FC: fully connected (layer); GRU: gated recurrent unit; KNN: K-nearest neighbors; LDA: linear discriminant analysis; LGB: light gradient boosting machine; LR: logistic regression; LSTM: long short-term memory; MLP: multilayer perceptron; NB: naive Bayes; NN: neural network; QDA: quadratic discriminant analysis; RF: random forest; SVM: support vector machine; XGBoost: Extreme Gradient Boosting.

In the included studies, classification was the most commonly used problem-solving strategy (45/46, 98%; Table 3). A variety of AI methods were used, with convolutional neural networks (CNNs) being the most popular (17/46, 37%), followed by random forest (14/46, 30%) and support vector machines (12/46, 26%). As shown in Figure 3, the adoption trends of these methods evolved over the years. The majority of the reviewed studies utilized AI for diagnosis and screening (44/46, 96%), while only a few focused on using wearable AI to predict sleep problems before they occurred (6/46, 13%). Approximately 31 studies reported a mean data set size of 59,647.4 (SD 133,284), with a range of 12-561,480. Open-source data were used in a small number of studies (7/46, 15%), whereas the majority relied on closed-source data (39/46, 85%). All studies (46/46, 100%) collected data using wearable technology, while 4 (9%) also incorporated self-reported questionnaires, and 2 (4%) utilized nonwearable technology, such as cell phones. The most commonly used data types for model development included breathing-related metrics (eg, respiratory rate and respiratory effort; 25/46, 54%), heart rate–related metrics (eg, heart rate, heart rate variability, and interbeat interval; 22/46, 48%), and body movement (activity levels; 17/46, 37%). A total of 23 studies reported the number of features used, ranging from 2 to 10,500, with an average of 497.7 (SD 2181).

The most commonly chosen reference standard was clinical assessment (35/46, 76%). As shown in Multimedia Appendix 7, PSG was the most frequently used clinical assessment method. To validate AI model performance, most studies used a train-test split and K-fold cross-validation (21/46, 46%). The most commonly used metric for evaluating AI algorithms was accuracy (34/46, 74%), followed by sensitivity (29/46, 63%) and specificity (27/46, 59%). Multimedia Appendices 8 and 9 provide details on AI model characteristics in each cited study.

**Table 3:** AI^a^ model characteristics.

Feature			Studies		References
**Problem-solving approach,** **n (%)**					
	Classification	45 (98)		[27-65,67-72]	
	Regression	13 (28)		[29,35,40,46-48,50,61-63,65,70,71]	
	Clustering	1 (2)		[66]	
**AI algorithms,** **n (%)**					
	Convolutional neural network	17 (37)		[27,29,30,32,33,36,44,45,47,52,58-60,63,65,67,72]	
	Random forest	14 (30)		[28,29,31,34,38-42,46,48,50,52,53]	
	Support vector machines	12 (26)		[31,34,41,42,46,48,52,53,69-72]	
	Long short-term memory	10 (22)		[29,41,49,52,54,57,61,63,65,67]	
	K-nearest neighbors	9 (20)		[34,39,41-43,50,52,64,72]	
	Naive Bayes	6 (13)		[39,41,42,48,50,64]	
	Multilayer perceptron	5 (11)		[29,38,46,52,68]	
	Artificial neural network	4 (9)		[51,55,62,64]	
	Decision trees	4 (9)		[39,41,48,50]	
	AdaBoost^b^	3 (7)		[41,43,48]	
	XGBoost^c^	3 (7)		[28,34,37]	
	Others <3	8 (17)		[29,31,41,46,48,52,63,66]	
	Not reported	2 (4)		[35,56]	
**Aim of AI algorithm,** **n (%)**					
	Diagnosis/screening	44 (96)		[27,29-65,67-72]	
	Prediction	6 (13)		[28,48,56,57,66,71]	
	Monitoring	1 (2)		[60]	
**Data set size**					
	Mean (SD)	59,647.4 (133,284)		[29,32,33,36-41,43-48,51,52,55-57,59,60,62-65,67,70]	
	Range	12-561,480		N/A^d^	
	Not reported, n (%)	15 (33)		[27,31,34,35,42,49,50,53,54,58,61,66,68,69,71]	
**Data source,** **n (%)**					
	Closed	39 (85)		[27,28,30-41,43-45,48-52,54,55,57-66,68-72]	
	Open	7 (15)		[29,42,46,47,53,56,67]	
**Data types,** **n (%)**					
	WD^e^ based	46 (100)		[27-72]	
	Self-reported	4 (9)		[37,66,67,69]	
	Non-WD based	2 (4)		[37,69]	
**Data input to AI algorithm,** **n (%)**					
	Respiration data	25 (54)		[29,32,36,37,40,41,43,46-48,52,54-61,63,65,68-71]	
	Heart rate	22 (48)		[28,33-35,37-39,41,44-47,50,51,57,59,61,62,64,68,70,71]	
	Body movement	17 (37)		[28,32,33,35,37,38,41,42,51,57,59,61,62,64,66,67,70]	
	Oxygen saturation	13 (28)		[29,32-35,37,40,51,52,54,62,69,71]	
	Acoustics data	3 (7)		[32,37,71]	
	Others <3	9 (20)		[27,30,31,37,49,53,59,67,72]	
**Number of features**					
	Mean (SD)	497.7 (2181)		[31,33,38-43,45-48,50,53,54,58,61-63,65,68,69,71]	
	Range	2-10,500		N/A	
	Not reported, n (%)	23 (50)		[27-30,32,34-37,44,49,51,52,55-57,59,60,64,66,67,70,72]	
**Reference standard,** **n (%)**					
	Clinical assessment	35 (76)		[29,30,33-40,43-54,56-59,61-63,65-67,69-71]	
	Wearable device	2 (4)		[27,55]	
	Context	3 (7)		[41,60,68]	
	Not reported	6 (13)		[27,28,31,32,42,72]	
**Type of validation,** **n (%)**					
	Train-test split	21 (46)		[27,28,32,33,35,36,41,44-47,51,54-56,58-62,64]	
	K-fold cross-validation	21 (46)		[29,31,33,37,39,40,42-45,48,50,52,57,63-65,67,68,71,72]	
	Leave-one-out cross-validation	6 (13)		[29,32,38,53,69,70]	
	Not reported	3 (7)		[30,34,66]	
	Others	6 (13)		[27,28,31,42,49,72]	
**Machine learning performance measures,** **n (%)**					
	Accuracy	34 (74)		[27,29,31-34,36,37,39-47,50-53,56-62,64,67,68,70-72]	
	Sensitivity (recall)	33 (72)		[27-29,31-35,37-54,56,57,59,62,63,67,70]	
	Specificity	27 (59)		[27-29,31-35,37-39,41-47,50-53,56,57,59,62,70]	
	*F*_1_-score	16 (35)		[27,39-42,44,45,48,49,54,58,61,63,65,67,69]	
	Positive predictive value (precision)	19 (41)		[27,28,33,35,38,40,41,43,46-49,51,54,59,62,63,67,69]	
	Cohen κ	11 (24)		[29,30,33,46,47,49,51,52,59,61,62]	
	Area under the curve	11 (24)		[28,35,40,42,46,47,51,58,59,62,67]	
	Negative predictive value	9 (20)		[28,33,35,38,43,46,47,51,62]	
	Area under the precision curve	6 (13)		[35,40,46,47,51,59]	
	Positive likelihood ratio	4 (9)		[33,35,51,62]	
	Negative likelihood ratio	4 (9)		[33,35,51,62]	
	Diagnostic odds ratio	1 (2)		[38]	
	Not reported	2 (4)		[55,66]	

**^a^**AI: artificial intelligence.

^b^AdaBoost: Adaptive Boosting.

^c^XGBoost: Extreme Gradient Boosting.

^d^N/A: not applicable.

^e^WD: wearable device.

## Discussion

### Principal Findings

This scoping review explored the features of wearable AI technology used for sleep disorders. Consistent with previous reviews [73,74], we observed a positive trend in the adoption of wearable technology, reflecting a growing interest in sleep disorder research. The majority of studies were conducted in Asia (19/46, 41%), nearly twice as many as those in North America (12/46, 26%) and Europe (13/46, 28%). The gap between Asia and North America-Europe may be multifaceted. Contributing factors could include regional differences in sleep patterns [75-79] and the availability and affordability of WDs in Asia. Most studies focused on the middle-aged population (mean age 45 years), reflecting the higher prevalence of sleep disorders such as insomnia, sleep apnea, and restless leg syndrome in this group [80].

Key findings include the dominance of wearable AI in sleep apnea research (39/46, 85%). This can be attributed to the high prevalence of sleep apnea [81], its detrimental health effects [82], the limitations of existing diagnostic techniques [83], and advancements in wearable technology, which have made sleep apnea a primary focus for the development of innovative wearable monitoring systems [25]. Commercially available WDs (30/46, 65%) were predominantly used due to their accessibility, affordability, and ease of use [84,85], reflecting a shift away from prototypes seen in previous studies [86]. Wrist-worn devices and accelerometer sensors were the most commonly utilized technologies (34/46, 74%), often combined with photoplethysmography sensors to enhance sleep staging accuracy [87]. Another key finding of this review is that, despite the availability of well-known sleep wearables such as the Actiwatch and Belun Ring, relatively few studies used these devices. This may be due to their high cost or their specialized design and marketing for specific sleep disorders.

More than two-thirds of the studies used AI for sleep disorder screening and diagnosis, highlighting its value as a diagnostic tool due to its scalability, ability to identify high-risk individuals, and capacity to detect sleep disorders from wearable sensor data [88,89]. CNNs were the most commonly used AI models (17/46, 37%), likely due to the nature of wearable data, which are collected from raw sensors and require extensive preprocessing, including feature engineering and data cleaning. CNNs are well-suited for this task as they excel in handling complex data, extracting key features, modeling nonlinear relationships, and performing effectively on large data sets [90]. As shown in Multimedia Appendix 7, from 2013 to 2023, there has been a growing diversity in AI algorithms, with CNN, long short-term memory, and random forest remaining the most commonly used. The increasing adoption of ensemble and hybrid AI methods suggests a trend toward enhancing model performance. Data for AI models were predominantly sourced from closed data sets (39/46, 85%), with studies either recruiting their own participants or utilizing precollected hospital data. This preference may stem from privacy and ethical concerns, as these data sets often contain sensitive personal and physiological information, requiring additional safeguards and regulatory compliance for public sharing. The primary data types used for AI model development included respiratory data (25/46, 54%), heart rate (22/46, 48%), and body movement (17/46, 37%), as these are crucial for identifying the underlying causes of sleep disorders [43,91,92]. Respiratory rate was frequently utilized due to its critical role in detecting sleep apnea, the primary focus of most studies. Clinical assessments, particularly PSG (28/46, 61%), were the most commonly used reference standards for validation. PSG involves placing multiple sensors to monitor brain and heart activity, eye movements, muscle activity, blood oxygen levels, breathing patterns, body movements, snoring, and other noises, making it a widely preferred method for its accuracy and comprehensive assessment in sleep studies. Half of the studies validated their AI models using train-test split and K-fold cross-validation methods. K-fold cross-validation is especially effective at capturing data variability and is well-suited for smaller data sets, which are common in wearable studies [93]. However, the train-test split method was equally utilized. This preference may stem from its simplicity, ease of implementation, unbiased performance estimation, flexibility with data set size, and alignment with established best practices.

### Comparison With Prior Work

Our findings align with previous reviews [73,74], which reported an increasing use of wearable technology in sleep disorders research. However, unlike prior reviews that highlighted a focus on prototypes [86], we observed a significant shift toward commercially available devices, driven by technological advancements and affordability. Consistent with earlier studies [18,94], wrist-worn devices were the most commonly used, likely due to their portability and cost-effectiveness. While accelerometer-based wearables remained prevalent [95], this review highlights an emerging trend of integrating additional sensors, such as photoplethysmography, to enhance accuracy—an aspect less evident in earlier reviews. Furthermore, the increasing adoption of ensemble and hybrid AI methods represents a recent development in wearable AI applications for sleep disorders.

### Strengths

This review comprehensively assessed wearable AI technologies for sleep disorders, offering insights into their applications, regional trends, and preferences for sensors and algorithms. A key strength of this review is its focus on noninvasive WDs deployed in studies. By including research spanning a decade (2013-2023), we captured evolving trends in wearable technology and AI methodologies. Additionally, an extensive search across 7 diverse databases (eg, MEDLINE, Embase, IEEE Xplore) encompassing psychological, biomedical, technological, and interdisciplinary research ensured a comprehensive analysis.

### Limitations

First, this scoping review focused solely on WDs worn on the body, excluding nonwearable, implanted, and handheld devices (such as smartphones and carry-on sensors); near-body sensors (eg, Bluetooth transmitters); and devices requiring clinical intervention. As a result, the generalizability of our findings to such devices may be limited. However, by narrowing the scope, we ensured a focused review of wearable AI applications that are accessible and user-friendly. Second, we excluded studies that examined AI applications for detecting sleep quality or sleep staging without directly addressing sleep disorders, as well as those forecasting the outcomes of interventions for sleep disorders. Future reviews could broaden the scope to include these areas, providing a more holistic understanding of wearable AI applications in sleep research. Third, only studies published in English were included, which may have led to the omission of relevant research in other languages. Fourth, this review focused solely on the features of WDs and AI models and did not evaluate the efficacy or performance of wearable AI, as this was beyond its scope. Systematic reviews and meta-analyses, which assess quality and validate performance, are needed for such evaluations. Fifth, the rapidly evolving nature of wearable AI technology may mean that some recent advancements were not captured. Frequent updates to scoping reviews and systematic reviews can ensure timely insights into this dynamic field.

### Future Directions

#### Practical Implications

To improve overall patient care and outcomes, AI applications in sleep disorders must extend beyond diagnosis and screening. While these areas are crucial, expanding AI use to include predicting sleep disorders, delivering personalized interventions or treatments, and providing tailored recommendations could unlock its full potential. Researchers should explore the capabilities of advanced models, such as large language models (LLMs), in sleep medicine. Investigating these areas will not only advance sleep medicine but also contribute to the refinement of LLMs, as their applications in health care are still evolving. A significant research gap remains, requiring thorough evaluation and validation, along with the active involvement of medical professionals in shaping the development and clinical implementation of these tools.

Despite extensive literature on significant differences in sleep patterns between males and females, most of the reviewed studies did not account for these variations. Notable differences include sleep duration, with females requiring approximately 20 minutes more sleep per night than males [96], and sleep architecture, as females generally exhibit a higher percentage of slow-wave sleep and spend more time in stage 3 non–rapid eye movement sleep than males [97]. Additionally, certain sleep disorders exhibit gender-based differences in prevalence; for example, obstructive sleep apnea is more common in males, whereas restless legs syndrome and insomnia are more prevalent in females [96,98,99]. Future studies should account for these gender differences and related factors when developing machine learning models for diagnosing, predicting, or monitoring sleep disorders using WDs. AI applications should incorporate gender-specific diagnostics, predictive analytics for disorder risk, and targeted interventions, such as personalized sleep hygiene recommendations or treatment efficacy monitoring. Gender data can also be leveraged in federated learning to develop globally resilient models. Addressing these variations ensures that AI-powered sleep disorder solutions are both equitable and effective. Gender-specific algorithms could enhance the accuracy and applicability of WDs, leading to improved personalized care. Prioritizing this aspect in both data collection and model training is essential to ensure fair and effective solutions for all users.

Notably, none of the AI models used in the included studies were integrated into the WDs themselves. Given current technological advancements, we recommend that major manufacturers incorporate AI modules within these devices using TinyML and federated learning. This approach would enable continuous monitoring and real-time alerts for irregular patterns, benefiting both patients and their care providers. These changes would not only provide manufacturers with a competitive edge but also increase acceptance rates among the general population and enhance self-awareness. Additionally, AI models heavily rely on data—the larger the data set, the better the model’s generalizability. This review noted that most studies used proprietary (closed-source) data sets, with only a few utilizing open-source data. To foster accessibility, collaboration, and innovation among researchers, there is a need for more open-source data sets. Such data sets not only enhance scientific integrity by enabling reproducibility and validation of findings but also support researchers with limited resources. This approach would encourage more interdisciplinary research and facilitate the development of more robust AI/machine learning models. Therefore, researchers are encouraged to publish their data sets in open-source databases while ensuring proper consent and thorough deidentification of data to protect privacy.

In the included studies, the ground truth for sleep disorders was primarily determined through clinical assessment, with PSG being the most commonly used method. While PSG remains the gold standard for sleep assessment, its complex setup and high costs limit its feasibility for regular testing, which is crucial for AI model optimization. Researchers should explore more flexible, accessible, and cost-effective alternatives for long-term monitoring, especially in nonclinical settings. This could include leveraging well-established standard devices or integrating automated scoring systems.

#### Research Implications

This review explored the general application of wearable AI in sleep disorders without conducting an in-depth performance evaluation. To thoroughly assess AI performance, systematic reviews and meta-analyses are needed. Each sleep disorder should have a dedicated systematic review analyzing the AI technologies proposed as solutions. Researchers could also investigate popular sleep-tracking devices such as Fitbit, Oura Ring, Whoop, and Garmin, comparing their accuracy and user acceptance in sleep monitoring. Further scoping and systematic reviews on sleep disorders will help researchers, wearable companies, and developers better identify the specific needs of their target population, particularly in relation to AI algorithms.

This review identified significant regional disparities in research trends. To foster collaboration and address global health needs, greater transparency in WD adoption across regions is essential. Establishing practical standards for WD development would enhance biosignal measurement accuracy, improve algorithmic performance, and advance research. Collaborative efforts are crucial to bridging these gaps and ensuring the global applicability of findings.

While sleep apnea is undeniably one of the most prevalent sleep disorders, this review found that relatively few studies focused on other significant conditions. Many sleep disorders remain underdiagnosed or misdiagnosed, leading to inadequate treatment and prolonged distress. Expanding research beyond sleep apnea would improve our understanding of sleep physiology and neurobiology, potentially driving breakthroughs in diagnosis and treatment for multiple conditions.

### Conclusions

Noninvasive wearable AI devices hold significant potential for detecting and monitoring sleep disorders. Our review highlights a growing global research trend in this area. However, to comprehensively assess the performance of wearable AI, further systematic reviews are needed to statistically synthesize study results. Additionally, more research should explore wearable AI applications beyond sleep apnea. Future AI developments should extend beyond diagnosis and screening to include predicting sleep disorders, delivering personalized interventions, and providing tailored recommendations. Advanced AI models, such as generative AI and LLMs, should be explored in line with current technological trends. Manufacturers should integrate these models into WDs to enhance functionality and user experience. Additionally, studies should provide sufficient details on findings and model architectures to facilitate comprehensive systematic reviews and meta-analyses.

## Appendix

Multimedia Appendix 1 PRISMA-ScR checklist.

Multimedia Appendix 2 Search strategy.

Multimedia Appendix 3 Data extraction form.

Multimedia Appendix 4 Characteristics of each included study.

Multimedia Appendix 5 Features of wearable devices.

Multimedia Appendix 6 Features of sensors of wearable devices.

Multimedia Appendix 7 Reference standards used for evaluating wearables per study.

Multimedia Appendix 8 Features of artificial intelligence algorithms.

Multimedia Appendix 9 Features of data used in artificial intelligence algorithms.

## Abbreviations

AI: artificial intelligence
CNN: convolutional neural network
HST: home sleep testing
IEC: International Electrotechnical Commission
PRISMA-ScR: Preferred Reporting Items for Systematic Reviews and Meta-Analyses extension for Scoping Reviews
PSG: polysomnography
WD: wearable device

## Data Availability

All data generated during this study are provided as multimedia appendices.
