# Supplementary material for: Wearable Artificial Intelligence for Sleep Disorders: Scoping Review
Source: J Med Internet Res. 2025 May 6;27:e65272. doi: 10.2196/65272 (PMC12093076; doi:10.2196/65272)
Supplement: Multimedia Appendix 2 [file jmir_v27i1e65272_app2.docx]

| Monday, May 30, 2022 6:45:26 AM Monday, May 30, 2022 6:39:40 AM |
| --- |

**Appendix 2: Search strategy**

Database(s): **Ovid MEDLINE(R) ALL**1946 to September 20, 2023
Search Strategy:

| **#** | **Searches** | **Results** |
| --- | --- | --- |
| 1 | exp Artificial Intelligence/ | 179456 |
| 2 | Artificial Intelligence.tw. | 32827 |
| 3 | exp Machine Learning/ | 60204 |
| 4 | "Machine Learning".tw. | 83556 |
| 5 | exp Deep Learning/ | 16593 |
| 6 | "Deep Learning".tw. | 45138 |
| 7 | "Supervised learning".tw. | 4959 |
| 8 | "unsupervised learning".tw. | 2175 |
| 9 | "reinforcement learning".tw. | 5580 |
| 10 | "Computer vision".tw. | 7265 |
| 11 | "Decision tree*".tw. | 14889 |
| 12 | "K-Nearest Neighbor*".tw. | 5109 |
| 13 | "Support vector machine*".tw. | 25484 |
| 14 | "convolutional neural network*".tw. | 23084 |
| 15 | "Recurrent neural network*".tw. | 4142 |
| 16 | "Artificial neural network*".tw. | 17085 |
| 17 | "deep neural network*".tw. | 9019 |
| 18 | "Naïve Bayes".tw. | 8 |
| 19 | "Naive Bayes".tw. | 3187 |
| 20 | "Fuzzy Logic".tw. | 2412 |
| 21 | "logistic regression".tw. | 403059 |
| 22 | "K-Means".tw. | 7054 |
| 23 | "Random Forest".tw. | 19964 |
| 24 | "Long Short-Term Memory Networks".tw. | 189 |
| 25 | "Linear Discriminant Analysis".tw. | 7346 |
| 26 | "Capsule Network*".tw. | 222 |
| 27 | "deep belief network".tw. | 316 |
| 28 | "Gradient Boost*".tw. | 5485 |
| 29 | AdaBoost.tw. | 1356 |
| 30 | "Multilayer Perceptron".tw. | 2585 |
| 31 | exp Wearable Electronic Devices/ | 19122 |
| 32 | wearable*.tw. | 25480 |
| 33 | "smart watch*".tw. | 226 |
| 34 | smartwatch*.tw. | 1024 |
| 35 | acceleromet*.tw. | 22131 |
| 36 | gyroscop*.tw. | 2464 |
| 37 | "inertial sensor".tw. | 1157 |
| 38 | "inertial measurement unit*".tw. | 2851 |
| 39 | smartband*.tw. | 22 |
| 40 | headband*.tw. | 363 |
| 41 | "head band*".tw. | 75 |
| 42 | wristband*.tw. | 774 |
| 43 | "wrist band*".tw. | 98 |
| 44 | "smart band*".tw. | 92 |
| 45 | bracelet*.tw. | 684 |
| 46 | Emotiv.tw. | 93 |
| 47 | NeuroSky.tw. | 19 |
| 48 | Muse.tw. | 651 |
| 49 | Fitbit.tw. | 1217 |
| 50 | Garmin.tw. | 267 |
| 51 | "Polar loop".tw. | 32 |
| 52 | Jawbone.tw. | 919 |
| 53 | Geneactiv.tw. | 188 |
| 54 | Empatica.tw. | 98 |
| 55 | Actigraph.tw. | 3613 |
| 56 | "Apple Watch".tw. | 293 |
| 57 | Amazfit.tw. | 5 |
| 58 | Actiwatch.tw. | 392 |
| 59 | "Mi Band".tw. | 43 |
| 60 | "Oura Ring".tw. | 36 |
| 61 | Vivosmart.tw. | 41 |
| 62 | "Microsoft Band".tw. | 22 |
| 63 | "Actiwave cardio".tw. | 3 |
| 64 | "MindWave Mobile".tw. | 3 |
| 65 | "Galaxy watch".tw. | 16 |
| 66 | Biobeam.tw. | 5 |
| 67 | "Belun Ring".tw. | 4 |
| 68 | "Belun Sleep System".tw. | 1 |
| 69 | "Wellue O2 ring".tw. | 0 |
| 70 | AirGo.tw. | 4 |
| 71 | BioStamp.tw. | 12 |
| 72 | "sleep disorder*".tw. | 24954 |
| 73 | "sleep disturbance*".tw. | 20943 |
| 74 | "sleep abnormalit*".tw. | 754 |
| 75 | "sleep apnea".tw. | 38665 |
| 76 | exp Sleep Apnea Syndromes/ | 43468 |
| 77 | exp "Sleep Initiation and Maintenance Disorders"/ | 17917 |
| 78 | insomnia.tw. | 28879 |
| 79 | Narcolepsy.tw. | 5223 |
| 80 | "Restless Legs Syndrome".tw. | 4582 |
| 81 | "Periodic Limb Movement Disorder".tw. | 300 |
| 82 | Parasomnias.tw. | 974 |
| 83 | Hypersomnia.tw. | 2163 |
| 84 | Bruxism.tw. | 3092 |
| 85 | 1 or 2 or 3 or 4 or 5 or 6 or 7 or 8 or 9 or 10 or 11 or 12 or 13 or 14 or 15 or 16 or 17 or 18 or 19 or 20 or 21 or 22 or 23 or 24 or 25 or 26 or 27 or 28 or 29 or 30 | 708230 |
| 86 | 31 or 32 or 33 or 34 or 35 or 36 or 37 or 38 or 39 or 40 or 41 or 42 or 43 or 44 or 45 or 46 or 47 or 48 or 49 or 50 or 51 or 52 or 53 or 54 or 55 or 56 or 57 or 58 or 59 or 60 or 61 or 62 or 63 or 64 or 65 or 66 or 67 or 68 or 69 or 70 or 71 | 67705 |
| 87 | 72 or 73 or 74 or 75 or 76 or 77 or 78 or 79 or 80 or 81 or 82 or 83 or 84 | 124658 |
| 88 | 85 and 86 and 87 | 118 |
| 89 | limit 88 to (english language and humans) | 87 |

Database(s): **Embase**1974 to 2023 Week 37
Search Strategy:

| **#** | **Searches** | **Results** |
| --- | --- | --- |
| 1 | exp Artificial Intelligence/ | 85897 |
| 2 | Artificial Intelligence.tw. | 39348 |
| 3 | exp Machine Learning/ | 415941 |
| 4 | "Machine Learning".tw. | 98628 |
| 5 | exp Deep Learning/ | 44236 |
| 6 | "Deep Learning".tw. | 52403 |
| 7 | "Supervised learning".tw. | 5519 |
| 8 | "unsupervised learning".tw. | 2415 |
| 9 | "reinforcement learning".tw. | 6247 |
| 10 | "Computer vision".tw. | 7846 |
| 11 | "Decision tree*".tw. | 20762 |
| 12 | "K-Nearest Neighbor*".tw. | 5973 |
| 13 | "Support vector machine*".tw. | 30517 |
| 14 | "convolutional neural network*".tw. | 27078 |
| 15 | "Recurrent neural network*".tw. | 4623 |
| 16 | "Artificial neural network*".tw. | 19874 |
| 17 | "deep neural network*".tw. | 10018 |
| 18 | "Naïve Bayes".tw. | 24 |
| 19 | "Naive Bayes".tw. | 3861 |
| 20 | "Fuzzy Logic".tw. | 2918 |
| 21 | "logistic regression".tw. | 583052 |
| 22 | "K-Means".tw. | 9384 |
| 23 | "Random Forest".tw. | 24624 |
| 24 | "Long Short-Term Memory Networks".tw. | 200 |
| 25 | "Linear Discriminant Analysis".tw. | 8832 |
| 26 | "Capsule Network*".tw. | 229 |
| 27 | "deep belief network".tw. | 362 |
| 28 | "Gradient Boost*".tw. | 6593 |
| 29 | AdaBoost.tw. | 1621 |
| 30 | "Multilayer Perceptron".tw. | 2882 |
| 31 | exp Wearable Electronic Devices/ | 8918 |
| 32 | wearable*.tw. | 27880 |
| 33 | "smart watch*".tw. | 346 |
| 34 | smartwatch*.tw. | 1257 |
| 35 | acceleromet*.tw. | 27820 |
| 36 | gyroscop*.tw. | 2441 |
| 37 | "inertial sensor".tw. | 1334 |
| 38 | "inertial measurement unit*".tw. | 3012 |
| 39 | smartband*.tw. | 22 |
| 40 | headband*.tw. | 526 |
| 41 | "head band*".tw. | 109 |
| 42 | wristband*.tw. | 1099 |
| 43 | "wrist band*".tw. | 180 |
| 44 | "smart band*".tw. | 99 |
| 45 | bracelet*.tw. | 1011 |
| 46 | Emotiv.tw. | 141 |
| 47 | NeuroSky.tw. | 22 |
| 48 | Muse.tw. | 1271 |
| 49 | Fitbit.tw. | 1842 |
| 50 | Garmin.tw. | 370 |
| 51 | "Polar loop".tw. | 38 |
| 52 | Jawbone.tw. | 1005 |
| 53 | Geneactiv.tw. | 270 |
| 54 | Empatica.tw. | 126 |
| 55 | Actigraph.tw. | 5430 |
| 56 | "Apple Watch".tw. | 448 |
| 57 | Amazfit.tw. | 7 |
| 58 | Actiwatch.tw. | 998 |
| 59 | "Mi Band".tw. | 57 |
| 60 | "Oura Ring".tw. | 56 |
| 61 | Vivosmart.tw. | 50 |
| 62 | "Microsoft Band".tw. | 26 |
| 63 | "Actiwave cardio".tw. | 4 |
| 64 | "MindWave Mobile".tw. | 5 |
| 65 | "Galaxy watch".tw. | 29 |
| 66 | Biobeam.tw. | 8 |
| 67 | "Belun Ring".tw. | 7 |
| 68 | "Belun Sleep System".tw. | 1 |
| 69 | "Wellue O2 ring".tw. | 0 |
| 70 | AirGo.tw. | 8 |
| 71 | BioStamp.tw. | 26 |
| 72 | "sleep disorder*".tw. | 42596 |
| 73 | "sleep disturbance*".tw. | 33947 |
| 74 | "sleep abnormalit*".tw. | 1191 |
| 75 | "sleep apnea".tw. | 64616 |
| 76 | exp Sleep Apnea Syndromes/ | 69423 |
| 77 | exp "Sleep Initiation and Maintenance Disorders"/ | 86461 |
| 78 | insomnia.tw. | 48509 |
| 79 | Narcolepsy.tw. | 8309 |
| 80 | "Restless Legs Syndrome".tw. | 7335 |
| 81 | "Periodic Limb Movement Disorder".tw. | 500 |
| 82 | Parasomnias.tw. | 1750 |
| 83 | Hypersomnia.tw. | 3684 |
| 84 | Bruxism.tw. | 3620 |
| 85 | 1 or 2 or 3 or 4 or 5 or 6 or 7 or 8 or 9 or 10 or 11 or 12 or 13 or 14 or 15 or 16 or 17 or 18 or 19 or 20 or 21 or 22 or 23 or 24 or 25 or 26 or 27 or 28 or 29 or 30 | 1082230 |
| 86 | 31 or 32 or 33 or 34 or 35 or 36 or 37 or 38 or 39 or 40 or 41 or 42 or 43 or 44 or 45 or 46 or 47 or 48 or 49 or 50 or 51 or 52 or 53 or 54 or 55 or 56 or 57 or 58 or 59 or 60 or 61 or 62 or 63 or 64 or 65 or 66 or 67 or 68 or 69 or 70 or 71 | 72766 |
| 87 | 72 or 73 or 74 or 75 or 76 or 77 or 78 or 79 or 80 or 81 or 82 or 83 or 84 | 238815 |
| 88 | 85 and 86 and 87 | 264 |
| 89 | limit 88 to (english language and humans) | 256 |
| 90 | limit 89 to "remove medline records" | 140 |

| **Database** | **Query** | **Results** |
| --- | --- | --- |
| **Scopus** | ( TITLE-ABS-KEY ( "artificial intelligence" OR "machine learning" OR "deep learning" OR "supervised learning" OR "unsupervised learning" OR "reinforcement learning" OR "decision tree" OR "k-nearest neighbor*" OR "support vector machine*" OR "recurrent neural network*" OR "convolutional neural network*" OR "artificial neural network*" OR "deep neural networks" OR "naïve bayes" OR "naive bayes" OR "fuzzy logic" OR "k-means" OR "random forest" OR "long short-term memory networks" OR "deep belief network" OR "gradient boost*" OR "adaboost" OR "multilayer perceptron" OR "ensemble learning" OR "linear discriminant analysis" ) AND TITLE-ABS-KEY ( wearable* OR "smart watch*" OR smartwatch* OR "smart band*" OR smartband* OR acceleromet* OR gyroscop* OR "inertial sensor" OR "inertial measurement unit*" OR headband* OR "head band*" OR "wrist band*" OR wristband* OR bracelet* OR "belun ring" OR "belun sleep system" OR "wellue o2 ring" OR airgo OR biostamp OR emotiv OR neurosky OR muse OR fitbit OR garmin OR "polar loop" OR jawbone OR geneactiv OR empatica OR actigraph OR "apple watch" OR amazfit OR actiwatch OR "mi band" OR "oura ring" OR vivosmart OR "microsoft band" OR "actiwave cardio" OR "mindwave mobile" OR "galaxy watch" OR biobeam ) AND TITLE-ABS-KEY ( "sleep disorder*" OR "sleep disturbance*" OR "sleep abnormalit*" OR "sleep apnea" OR "sleep hypopnea" OR insomnia OR narcolepsy OR "restless legs syndrome" OR "periodic limb movement disorder" OR parasomnias OR hypersomnia OR bruxism ) ) AND ( LIMIT-TO ( LANGUAGE , "english" ) ) | 297 |
| **IEEE Xplore** | ("Abstract":"Artificial Intelligence" OR "Abstract":"Machine Learning" OR "Abstract":"Deep Learning" OR "Abstract":"supervised learning" OR "Abstract":"unsupervised learning" OR "Abstract":"reinforcement learning" OR "Abstract":"Decision tree" OR "Abstract":"K-Nearest Neighbor" OR "Abstract":"Support vector machine" OR "Abstract":"Recurrent neural network" OR "Abstract":"convolutional neural network" OR "Abstract":"Artificial neural network" OR "Abstract":"Deep neural networks" OR "Abstract":"Naïve Bayes" OR "Abstract":"Naive Bayes" OR "Abstract":"Fuzzy Logic" OR "Abstract":"K-Means" OR "Abstract":"Random Forest" OR "Abstract":"Long Short-Term Memory Networks" OR "Abstract":"deep belief network" OR "Abstract":"Gradient Boost*" OR "Abstract":"AdaBoost" OR "Abstract":"Multilayer Perceptron" OR "Abstract":"Ensemble learning" OR "Abstract":"Linear Discriminant Analysis") AND ("Abstract":wearable* OR "Abstract":"smart watch*" OR "Abstract":smartwatch* OR "Abstract":"smart band*" OR "Abstract":smartband* OR "Abstract":acceleromet OR "Abstract":gyroscop OR "Abstract":"inertial sensor" OR "Abstract":"inertial measurement unit" OR "Abstract":headband* OR "Abstract":"head band" OR "Abstract":"wrist band*" OR "Abstract":wristband* OR "Abstract":bracelet OR "Abstract":"Belun Ring" OR "Abstract":"Belun Sleep System" OR "Abstract":"Wellue O2 ring" OR "Abstract":AirGo OR "Abstract":BioStamp OR "Abstract":Emotiv OR "Abstract":NeuroSky OR "Abstract":Muse OR "Abstract":Fitbit OR "Abstract":Garmin OR "Abstract":"Polar loop" OR "Abstract":Jawbone OR "Abstract":Geneactiv OR "Abstract":Empatica OR "Abstract":Actigraph OR "Abstract":"Apple Watch" OR "Abstract":Amazfit OR "Abstract":Actiwatch OR "Abstract":"Mi Band" OR "Abstract":"Oura Ring" OR "Abstract":Vivosmart OR "Abstract":"Microsoft Band" OR "Abstract":"Actiwave cardio" OR "Abstract":"MindWave Mobile" OR "Abstract":"Galaxy watch" OR "Abstract":Biobeam) AND ("Abstract":"sleep disorders" OR "Abstract":"sleep disturbance" OR "Abstract":"sleep abnormality" OR "Abstract":"sleep apnea" OR "Abstract":"sleep hypopnea" OR "Abstract":insomnia OR "Abstract":Narcolepsy OR "Abstract":"Restless Legs Syndrome" OR "Abstract":"Periodic Limb Movement Disorder" OR "Abstract":Parasomnias OR "Abstract":Hypersomnia OR "Abstract":Bruxism) | 41 |
| **ACM Digital library** | [[Full Text: "artificial intelligence"] OR [Full Text: "machine learning"] OR [Full Text: "deep learning"] OR [Full Text: "supervised learning"] OR [Full Text: "unsupervised learning"] OR [Full Text: "reinforcement learning"] OR [Full Text: "decision tree"] OR [Full Text: "k-nearest neighbor*"] OR [Full Text: "support vector machine*"] OR [Full Text: "recurrent neural network*"] OR [Full Text: "convolutional neural network*"] OR [Full Text: "artificial neural network*"] OR [Full Text: "deep neural networks"] OR [Full Text: "naïve bayes"] OR [Full Text: "naive bayes"] OR [Full Text: "fuzzy logic"] OR [Full Text: "k-means"] OR [Full Text: "random forest"] OR [Full Text: "long short-term memory networks"] OR [Full Text: "deep belief network"] OR [Full Text: "gradient boost*"] OR [Full Text: "adaboost"] OR [Full Text: "multilayer perceptron"] OR [Full Text: "ensemble learning"] OR [Full Text: "linear discriminant analysis"]] AND [[Full Text: wearable*] OR [Full Text: "smart watch*"] OR [Full Text: smartwatch*] OR [Full Text: "smart band*"] OR [Full Text: smartband*] OR [Full Text: acceleromet*] OR [Full Text: gyroscop*] OR [Full Text: "inertial sensor"] OR [Full Text: "inertial measurement unit*"] OR [Full Text: headband*] OR [Full Text: "head band*"] OR [Full Text: "wrist band*"] OR [Full Text: wristband*] OR [Full Text: bracelet*] OR [Full Text: "belun ring"] OR [Full Text: "belun sleep system"] OR [Full Text: "wellue o2 ring"] OR [Full Text: airgo] OR [Full Text: biostamp] OR [Full Text: emotiv] OR [Full Text: neurosky] OR [Full Text: muse] OR [Full Text: fitbit] OR [Full Text: garmin] OR [Full Text: "polar loop"] OR [Full Text: jawbone] OR [Full Text: geneactiv] OR [Full Text: empatica] OR [Full Text: actigraph] OR [Full Text: "apple watch"] OR [Full Text: amazfit] OR [Full Text: actiwatch] OR [Full Text: "mi band"] OR [Full Text: "oura ring"] OR [Full Text: vivosmart] OR [Full Text: "microsoft band"] OR [Full Text: "actiwave cardio"] OR [Full Text: "mindwave mobile"] OR [Full Text: "galaxy watch"] OR [Full Text: biobeam]] AND [[Abstract: "sleep disorder*"] OR [Abstract: "sleep disturbance*"] OR [Abstract: "sleep abnormalit*"] OR [Abstract: "sleep apnea"] OR [Abstract: "sleep hypopnea"] OR [Abstract: insomnia] OR [Abstract: narcolepsy] OR [Abstract: "restless legs syndrome"] OR [Abstract: "periodic limb movement disorder"] OR [Abstract: parasomnias] OR [Abstract: hypersomnia] OR [Abstract: bruxism]] | 24 |
| **Google Scholar** | ("Artificial Intelligence" OR "Machine Learning" OR "Deep Learning" OR "Decision tree" OR "K-Nearest Neighbor*" OR "Support vector machine*") AND (wearable* OR smartwatch* OR Muse OR Fitbit OR Empatica OR wristband* OR bracelet* OR "Belun Ring" OR "Oura Ring") AND ("sleep disorder*" OR "sleep apnea" OR insomnia) | 100 |
