# Supplementary material for: Wearable Artificial Intelligence for Sleep Disorders: Scoping Review
Source: J Med Internet Res. 2025 May 6;27:e65272. doi: 10.2196/65272 (PMC12093076; doi:10.2196/65272)
Supplement: Multimedia Appendix 3 [file jmir_v27i1e65272_app3.docx]

| **Extracted data** | **Definition** |
| --- | --- |
| **Study Characteristics** |  |
| Author | The first author of the study. |
| Year of publication | The year in which the study was published. |
| Country of publication | The country where the study was published. |
| Type of publication | The venue where the study was published: peer-reviewed journal articles, book chapters, dissertations, or conference proceedings |
| **Wearable devices characteristics** |  |
| Status of the wearable device | Is the wearable device a prototype (non-commercial) or is it an already available commercial device (e.g., Fitbit, apple watch)? |
| Name of the wearable device | What is the name of the wearable device (e.g., Fitbit, Empatica, Apply Watch, ActiWatch, etc..)? |
| Type of the wearable device | In which form the wearable device available (e.g., smart band, smart watch, smart glasses, smart clothes, smart socks, smart shoes, etc)? |
| Placement of the wearable device | Where the wearable device is worn during the experiment in paper or normally (wrist, chest, head, ears, forehead, eyes, fingers, foot, etc..)? |
| Measured biosignals | What are the biosignals measured by the wearable device (heart rate, EEG, ECG, step counts, body temperature, blood pressure, etc..)? |
| Sensors | What are the sensors embedded in the wearable device (Photoplethysmogram, accelerator, Gyroscope)? |
| Sensing approach | Does the wearable device collect the data with the user’s input (i.e., manually adding data; participatory approach) or without the user’s input (i.e., automatic mechanisms for collecting data; opportunistic approach)? |
| Status of the wearable device | Is the wearable device a prototype (non-commercial) or is it an already available commercial device (e.g., Fitbit, apple watch)? |
| Name of the wearable device | What is the name of the wearable device (e.g., Fitbit, Empatica, ApplyWatch, ActiWatch, etc..)? |
| Type of the wearable device | In which form the wearable device available (e.g., smart band, smart watch, smart glasses, smart clothes, smart socks, smart shoes, etc)? |
| Placement of the wearable device | Where the wearable device is worn during the experiment in paper or normally (wrist, chest, head, ears, forehead, eyes, fingers, foot, etc..)? |
| Measured biosignals | What are the biosignals measured by the wearable device (heart rate, EEG, ECG, step counts, body temperature, blood pressure, etc..)? |
| Sensors | What are the sensors embedded in the wearable device (Photoplethysmogram, accelerator, Gyroscope)? |
| Sensing approach | Does the wearable device collect the data with the user’s input (i.e., manually adding data; participatory approach) or without the user’s input (i.e., automatic mechanisms for collecting data; opportunistic approach)? |
| **AI** **Characteristics** |  |
| Problem solving approaches | What is the problem-solving approach that the algorithm follows (Classification, regression)? |
| AI algorithm used | What are the AI algorithms/models (e.g., RF, SVM, ANN, CNN, RNN, DNN, k-NN, MLP, DBN, DBM, DPN BN, CRT, DT, LASSO, LR, MFA, MLR, MDL, NB, NN, NSC, RBFN) used in the paper? |
| Aim of AI algorithm | What was the algorithm used for (diagnosis, screening, monitoring, treatment, prevention, etc.))? |
| Dataset size | What is the dataset size used for developing (training & testing) the algorithm? |
| Data sources | What is the source of data that was used for developing the algorithms: Closed source (collected by authors), Open source (public databases)? |
| Data types | What is the type of data (e.g., WD-based data, self-reported data, non-WD-based data) that was used for developing the algorithm? |
| Data input to AI algorithm | What is the data that was used for developing the algorithm? |
| Number of features | How many features of the collected data are used to train AI model? |
| Type of validation | What is the approach that was used to validate the developed algorithm (e.g., Training-test split, K-fold cross-validation, Nested Cross-Validation, Leave One Out cross-validation, Apparent validation, external validation)? |
| Performance measures used | What are the measures used to assess the performance of the algorithm (accuracy, sensitivity (recall), specificity, precision, AUC, etc...)? |
| Number of participants | What is the number of participants from which the data was collected? |
| Mean age (range) | What is the mean/range age of the participants? |
| Female percentage | What is the female percentage of the participants? |
| Targeted health condition | What is the health condition/disease that the AI-based wearable device targeted (e.g., sleep apnea or sleep hypopnea, insomnia, Narcolepsy, Restless Legs Syndrome, Periodic Limb Movement Disorder, Parasomnias, Hypersomnia, Bruxism? |
| Severity classification index | What index is commonly used to divide subjects into classification groups or labels, such as how the Apnea-Hypopnea Index (AHI) categorizes different levels of apnea into normal, mild-moderate, and severe groups? |
| Reference standard (Gold standard test) | How the actual status (e.g., diagnosis) of the user was confirmed (questionnaire (PHQ-9), interview, test, etc...)?  How were the participants validated at the time of requirement that they have this disorder? |
