# Supplementary material for: Wearable Artificial Intelligence for Sleep Disorders: Scoping Review
Source: J Med Internet Res. 2025 May 6;27:e65272. doi: 10.2196/65272 (PMC12093076; doi:10.2196/65272)
Supplement: Multimedia Appendix 6 [file jmir_v27i1e65272_app6.docx]

**Multimedia Appendix 6: Features of sensors of wearable devices**

| **Study [Ref]** | **Sensors** | **Sensing approach** | **Measured biosignals** |
| --- | --- | --- | --- |
| Benedetti [1] | ACC, PPG | Opportunistic approach | Activity measures, cardiovascular measures, oxygenation measures, sleep measures |
| Chang [2] | ACC, ECG | Opportunistic approach | Activity measures, cardiac measures, sleep measures |
| Chen [3] | PPG | Opportunistic approach | Cardiovascular measures, oxygenation measures |
| Chen [4] | ACC | Opportunistic approach | Activity measures, sleep measures |
| Fallmann [5] | ACC | Opportunistic approach | Activity measures, sleep measures |
| Fedorin [6] | ACC, PPG, light sensor | Opportunistic approach | Activity measures, cardiovascular measures, light exposure, oxygenation measures, sleep measures |
| Fedorin [7] | ACC, PPG | Opportunistic approach | Activity measures, cardiovascular measures, oxygenation measures, sleep measures |
| Ganglberger [8] | ACC | Opportunistic approach | Activity measures, sleep measures |
| Gu [9] | ACC, Oximeter, PPG | Opportunistic approach | Activity measures, cardiovascular measures, oxygenation measures, sleep measures |
| Hafezi [10] | ACC | Opportunistic approach | Activity measures, sleep measures |
| Hafezi [11] | ACC | Opportunistic approach | Activity measures, sleep measures |
| Hung [12] | ACC, ECG | NR | Activity measures, cardiac measures, sleep measures |
| Jeon [13] | ACC, gyroscope, magnetometer | Opportunistic approach | Activity measures, environmental measures, motion measures, sleep measures |
| Jeon [14] | ACC, PPG | Opportunistic approach | Activity measures, cardiovascular measures, oxygenation measures, sleep measures |
| Ji [15] | ACC, ECG | Opportunistic approach | Activity measures, cardiac measures, sleep measures |
| Kanal [16] | ACC, PPG | Opportunistic approach | Activity measures, cardiovascular measures, oxygenation measures, sleep measures |
| Kim [17] | ACC | Opportunistic approach | Activity measures, sleep measures |
| Kristiansen [18] | ACC | Opportunistic approach | Activity measures, sleep measures |
| Kristiansen [19] | ACC | Opportunistic approach, | Activity measures, sleep measures |
| Kusmakar [20] | ACC, light sensor | Opportunistic, participatory | Activity measures, light exposure,sleep measures |
| Kwon [21] | EEG, EOG, EMG | Opportunistic approach | Brain activity, eye movement, muscle activity |
| Le [22] | ECG, VCG | Opportunistic approach | Cardiac measures, cardiac electrical activity |
| McClure [23] | ACC, gyroscope | Opportunistic approach | Activity measures, motion measures, sleep measures |
| Papini [24] | ACC, PPG | Opportunistic approach | Activity measures, cardiovascular measures, oxygenation measures, sleep measures |
| Park [25] | ACC | Opportunistic approach | Activity measures, sleep measures |
| Petrenko [26] | ACC | Opportunistic approach | Activity measures, sleep measures |
| Rani [27] | ACC, light sensor | Opportunistic approach | Activity measures, light exposure, sleep measures |
| Raschellà [28] | ACC, light sensor | Opportunistic approach | Activity measures, light exposure, sleep measures |
| Rossi [29] | ACC, air pressure sensor | Opportunistic approach | Activity measures, acoustic data, sleep measures, |
| Ryser [30] | ACC | Opportunistic approach | Activity measures, sleep measures |
| Selvaraj [31] | ACC, ECG | Opportunistic approach | Activity measures, cardiac measures, sleep measures |
| Shen [32] | PPG | Opportunistic approach | Cardiovascular measures, oxygenation measures |
| Strumpf [33] | ACC, PPG | Opportunistic approach | Activity measures, cardiovascular measures, oxygenation measures, sleep measures |
| Tsouti [34] | Thoracic sensor, abdominal sensor | Opportunistic approach | Respiratory data |
| Van [35] | ACC, ECG | Opportunistic approach | Activity measures, cardiac measures, sleep measures |
| Wang [36] | PPG | Opportunistic approach | Cardiovascular measures, oxygenation measures |
| Wang [37] | ECG | Opportunistic approach | Cardiac measures |
| Wu [38] | ACC, Oximeter | Opportunistic approach | Activity measures, oxygenation measures, sleep measures |
| Wu [39] | PPG | Opportunistic approach | Cardiovascular measures, oxygenation measures |
| Xu [40] | Light sensor | Opportunistic approach | Oxygenation measures |
| Yeh [41] | ACC, PPG | Participatory approach | Activity measures, cardiovascular measures, oxygenation measures, sleep measures |
| Yeo [42] | ECG | Opportunistic approach | Cardiac measures |
| Yeo [43] | ECG | Opportunistic approach | Cardiac measures |
| Yüzer [44] | ACC | Opportunistic approach | Activity measures, sleep measures |
| Zhang [45] | Respiratory sensor | Opportunistic approach | Respiratory data |
| Zhou [46] | ACC, PPG | Opportunistic approach | Activity measures, cardiovascular measures, oxygenation measures, sleep measures |
| ACC; Accelerometer, ECG: Electrocardiogram, EEG: Electroencephalogram, PPG: photoplethysmogram, NR: Not reported | | | |

1. Benedetti, D., et al., *Obstructive Sleep Apnoea Syndrome Screening Through Wrist-Worn Smartbands: A Machine-Learning Approach.* Nat Sci Sleep, 2022. **14**: p. 941-956.

2. Chang, H.C., et al., *Portable Sleep Apnea Syndrome Screening and Event Detection Using Long Short-Term Memory Recurrent Neural Network.* Sensors (Basel), 2020. **20**(21).

3. Chen, M., et al., *Information-Based Similarity of Ordinal Pattern Sequences as a Novel Descriptor in Obstructive Sleep Apnea Screening Based on Wearable Photoplethysmography Bracelets.* Biosensors (Basel), 2022. **12**(12).

4. Chen, X., et al., *ApneaDetector: Detecting Sleep Apnea with Smartwatches.* Proc. ACM Interact. Mob. Wearable Ubiquitous Technol., 2021. **5**(2): p. Article 59.

5. Fallmann, S. and L. Chen. *Detecting Chronic Diseases from Sleep-Wake Behaviour and Clinical Features*. in *2018 5th International Conference on Systems and Informatics (ICSAI)*. 2018.

6. Fedorin, I. and K. Slyusarenko, *Consumer Smartwatches As a Portable PSG: LSTM Based Neural Networks for a Sleep-Related Physiological Parameters Estimation.* Annu Int Conf IEEE Eng Med Biol Soc, 2021. **2021**: p. 849-452.

7. Fedorin, I., K. Slyusarenko, and M. Nastenko, *Respiratory events screening using consumer smartwatches*. 2020. 25-28.

8. Ganglberger, W., et al., *Sleep apnea and respiratory anomaly detection from a wearable band and oxygen saturation.* Sleep Breath, 2022. **26**(3): p. 1033-1044.

9. Gu, W., et al., *Belun Ring Platform: a novel home sleep apnea testing system for assessment of obstructive sleep apnea.* J Clin Sleep Med, 2020. **16**(9): p. 1611-1617.

10. Hafezi, M., et al., *Sleep Apnea Severity Estimation From Tracheal Movements Using a Deep Learning Model.* IEEE Access, 2020. **8**: p. 22641-22649.

11. Hafezi, M., et al. *Sleep Apnea Severity Estimation from Respiratory Related Movements Using Deep Learning*. in *2019 41st Annual International Conference of the IEEE Engineering in Medicine and Biology Society (EMBC)*. 2019.

12. Hung, P.D., *Central Sleep Apnea Detection Using an Accelerometer*, in *Proceedings of the 1st International Conference on Control and Computer Vision*. 2018, Association for Computing Machinery: Singapore, Singapore. p. 106–111.

13. Jeon, S., Y.S. Lee, and S.H. Son, *Cascade Windows-Based Multi-Stream Convolutional Neural Networks Framework for Early Detecting In-Sleep Stroke Using Wristbands.* IEEE Access, 2023. **11**: p. 84944-84956.

14. Jeon, Y., K. Heo, and S.J. Kang, *Real-Time Sleep Apnea Diagnosis Method Using Wearable Device without External Sensors*. 2020. 1-5.

15. Ji, X., et al., *Airline Point-of-Care System on Seat Belt for Hybrid Physiological Signal Monitoring.* Micromachines, 2022. **13**(11): p. 1880.

16. Kanal, V., et al., *APSEN: Pre-screening Tool for Sleep Apnea in a Home Environment*. 2016.

17. Kim, W.P., et al., *Machine Learning-Based Prediction of Attention-Deficit/Hyperactivity Disorder and Sleep Problems With Wearable Data in Children.* JAMA Netw Open, 2023. **6**(3): p. e233502.

18. Kristiansen, S., et al., *Machine Learning for Sleep Apnea Detection with Unattended Sleep Monitoring at Home.* ACM Trans. Comput. Healthcare, 2021. **2**(2): p. Article 14.

19. Kristiansen, S., et al., *A clinical evaluation of a low-cost strain gauge respiration belt and machine learning to detect sleep apnea.* Smart Health, 2023. **27**: p. 100373.

20. Kusmakar, S., et al., *A machine learning model for multi-night actigraphic detection of chronic insomnia: Development and validation of a pre-screening tool.* Royal Society Open Science, 2021. **8**(6).

21. Kwon, S., et al., *At-home wireless sleep monitoring patches for the clinical assessment of sleep quality and sleep apnea.* Science Advances, 2023. **9**(21): p. eadg9671.

22. Le, T.Q., et al., *Wireless Wearable Multisensory Suite and Real-Time Prediction of Obstructive Sleep Apnea Episodes.* IEEE J Transl Eng Health Med, 2013. **1**: p. 2700109.

23. McClure, K., et al., *Classification and Detection of Breathing Patterns with Wearable Sensors and Deep Learning.* Sensors (Basel), 2020. **20**(22).

24. Papini, G.B., et al., *Wearable monitoring of sleep-disordered breathing: estimation of the apnea-hypopnea index using wrist-worn reflective photoplethysmography.* Sci Rep, 2020. **10**(1): p. 13512.

25. Park, S., et al., *Clustering Insomnia Patterns by Data From Wearable Devices: Algorithm Development and Validation Study.* JMIR Mhealth Uhealth, 2019. **7**(12): p. e14473.

26. Petrenko, A. *Breathmonitor: Sleep Apnea Mobile Detector*. in *2020 IEEE 2nd International Conference on System Analysis & Intelligent Computing (SAIC)*. 2020.

27. Rani, S., et al., *Differentiating acute from chronic insomnia with machine learning from actigraphy time series data.* Front Netw Physiol, 2022. **2**: p. 1036832.

28. Raschellà, F., et al., *Actigraphy Enables Home Screening of Rapid Eye Movement Behavior Disorder in Parkinson's Disease.* Ann Neurol, 2023. **93**(2): p. 317-329.

29. Rossi, M., et al., *SLEEP-SEE-THROUGH: Explainable Deep Learning for Sleep Event Detection and Quantification From Wearable Somnography.* IEEE J Biomed Health Inform, 2023. **27**(7): p. 3129-3140.

30. Ryser, F., et al., *Respiratory analysis during sleep using a chest-worn accelerometer: A machine learning approach.* Biomedical Signal Processing and Control, 2022. **78**: p. 104014.

31. Selvaraj, N. and R. Narasimhan, *Automated prediction of the apnea-hypopnea index using a wireless patch sensor.* 2014 36th Annual International Conference of the IEEE Engineering in Medicine and Biology Society, EMBC 2014, 2014. **2014**: p. 1897-900.

32. Shen, Q., et al., *Multitask Residual Shrinkage Convolutional Neural Network for Sleep Apnea Detection Based on Wearable Bracelet Photoplethysmography.* IEEE Internet of Things Journal, 2022. **9**(24): p. 25207-25222.

33. Strumpf, Z., et al., *Belun Ring (Belun Sleep System BLS-100): Deep learning-facilitated wearable enables obstructive sleep apnea detection, apnea severity categorization, and sleep stage classification in patients suspected of obstructive sleep apnea.* Sleep Health, 2023. **9**(4): p. 430-440.

34. Tsouti, V., et al., *Development of an automated system for obstructive sleep apnea treatment based on machine learning and breath effort monitoring.* Microelectronic Engineering, 2020. **231**: p. 111376.

35. Van Steenkiste, T., et al., *Portable Detection of Apnea and Hypopnea Events Using Bio-Impedance of the Chest and Deep Learning.* IEEE Journal of Biomedical and Health Informatics, 2020. **PP**: p. 1-1.

36. Wang, S., et al., *Machine Learning Assisted Wearable Wireless Device for Sleep Apnea Syndrome Diagnosis.* Biosensors, 2023. **13**(4): p. 483.

37. Wang, Z., et al., *Single-lead ECG based multiscale neural network for obstructive sleep apnea detection.* Internet of Things, 2022. **20**: p. 100613.

38. Wu, H.T., et al., *Phenotype-Based and Self-Learning Inter-Individual Sleep Apnea Screening With a Level IV-Like Monitoring System.* Front Physiol, 2018. **9**: p. 723.

39. Wu, S., et al., *Sleep apnea screening based on Photoplethysmography data from wearable bracelets using an information-based similarity approach.* Computer Methods and Programs in Biomedicine, 2021. **211**: p. 106442.

40. Xu, Y., et al., *Comparative study of a wearable intelligent sleep monitor and polysomnography monitor for the diagnosis of obstructive sleep apnea.* Sleep Breath, 2023. **27**(1): p. 205-212.

41. Yeh, E., et al., *Detection of obstructive sleep apnea using Belun Sleep Platform wearable with neural network-based algorithm and its combined use with STOP-Bang questionnaire.* PLoS One, 2021. **16**(10): p. e0258040.

42. Yeo, M., et al., *Respiratory Event Detection During Sleep Using Electrocardiogram and Respiratory Related Signals: Using Polysomnogram and Patch-Type Wearable Device Data.* IEEE J Biomed Health Inform, 2022. **26**(2): p. 550-560.

43. Yeo, M., et al., *Robust Method for Screening Sleep Apnea With Single-Lead ECG Using Deep Residual Network: Evaluation With Open Database and Patch-Type Wearable Device Data.* IEEE Journal of Biomedical and Health Informatics, 2022. **26**(11): p. 5428-5438.

44. Yüzer, A.H., et al., *A different sleep apnea classification system with neural network based on the acceleration signals.* Applied Acoustics, 2020. **163**: p. 107225.

45. Zhang, H., et al., *Long-Term Sleep Respiratory Monitoring by Dual-Channel Flexible Wearable System and Deep Learning-Aided Analysis.* IEEE Transactions on Instrumentation and Measurement, 2023. **72**: p. 1-9.

46. Zhou, G., et al., *Automatic monitoring of obstructive sleep apnea based on multi-modal signals by phone and smartwatch.* Annu Int Conf IEEE Eng Med Biol Soc, 2023. **2023**: p. 1-4.
