# Supplementary figures and images for: Wearable Artificial Intelligence for Sleep Disorders: Scoping Review
Source: J Med Internet Res. 2025 May 6;27:e65272. doi: 10.2196/65272 (PMC12093076; doi:10.2196/65272)

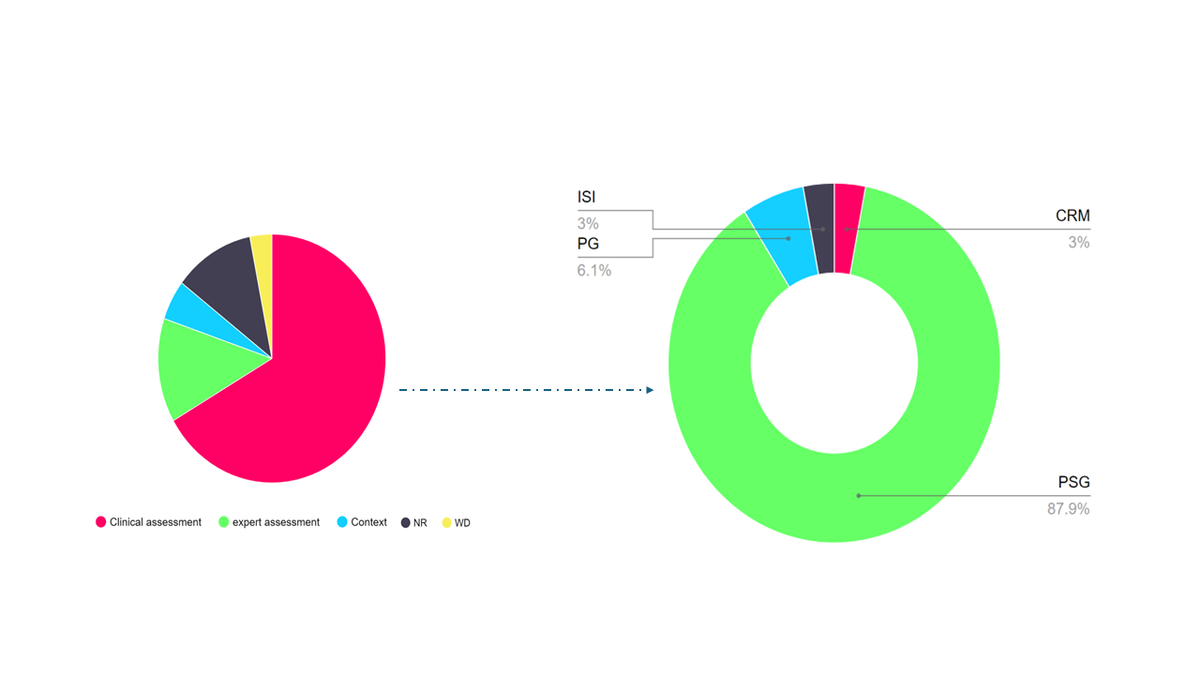

Supplement: Multimedia Appendix 7 [file jmir_v27i1e65272_app7.png]
