# Supplementary material for: Wearable Artificial Intelligence for Sleep Disorders: Scoping Review
Source: J Med Internet Res. 2025 May 6;27:e65272. doi: 10.2196/65272 (PMC12093076; doi:10.2196/65272)
Supplement: Multimedia Appendix 8 [file jmir_v27i1e65272_app8.docx]

**Multimedia Appendix 7: Features of AI algorithms**

| **Study [Ref]** | **Problem solving approaches** | **AI algorithms** | **Aim of AI algorithm** | **Type of validation** | **ML Performance measures** |
| --- | --- | --- | --- | --- | --- |
| Benedetti [1] | Classification | MLP, RF | diagnosis/ screening | LOOCV | sensitivity, specificity, positive predictive values (PPV), negative predictive values (NPV), diagnostic odds ratio (DOR) |
| Chang [2] | Classification | LSTM | diagnosis/ screening | Training-test split | Sensitivity, Precision, F1 score |
| Chen [3] | Classification | DT, KNN, NB, RF | diagnosis/ screening | K-fold CV | accuracy, sensitivity, specificity, F1 score |
| Chen [4] | Classification, regression | ABT, DT,LR, NB, RF, SVM | diagnosis/ screening, prediction | K-fold CV | precision, recall, F1-score |
| Fallmann [5] | Classification | LSTM, CNN | diagnosis/ screening | K-fold CV | accuracy, precision, recall, F1-score and AUC. |
| Fedorin [6] | classification | LSTM | diagnosis/ screening | External | precision, recall,F1 score, Cohen’s Kappa |
| Fedorin [7] | Classification, Regression | LSTM | diagnosis | Training-test split | accuracy, F1 score and Cohen’s kappa |
| Ganglberger [8] | Classification, Regression | RF | diagnosis/ screening | K-fold CV | ROC AUC, PRC AUC, accuracy, sensitivity, precision, F1 score |
| Gu [9] | Classification, Regression | ANN | diagnosis/ screening | Training-test split | accuracy, sensitivity, specificity, positive predictive value, negative predictive value, LR+, LR−, Cohen’s kappa, and area under the receiver operator curve |
| Hafezi [10] | Classification, Regression | CNN, LSTM,FC | diagnosis/ screening | K-fold CV | F1 score, Precision, Recall |
| Hafezi [11] | Classification, Regression | CNN, LSTM | diagnosis/ screening | K-fold CV | F1-score |
| Hung [12] | classification | MLP | diagnosis/ screening | K-fold CV | accuracy |
| Jeon [13] | Classification | CNN | diagnosis | Training-test split | Accuracy, sensitivity, specificity, F1 score, Precision |
| Jeon [14] | Classification | ANN, KNN, NB | diagnosis/ screening | K-fold CV, training-test split | accuracy |
| Ji [15] | Classification | AB, BP, DT, KNN, LSTM, NB, QDA, RF, SVM | diagnosis/ screening | Training-test split | Accuracy, sensitivity, specificity, F1 score, positive predictive value |
| Kanal [16] | classification | KNN, SVM, CNN | diagnosis/ screening | K-fold CV | Accuracy |
| Kim [17] | Classification | RF, XGB, LGB | Prediction | Training-test split | sensitivity, specificity, AUC, PPV, NPV |
| Kristiansen [18] | Classification | CNN, GRU, KNN, LSTM, MLP, RF, SVM | diagnosis/ screening | K-fold CV | Cohens Kappa, Accuracy, sensitivity, specificity, |
| Kristiansen [19] | Classification, regression | CNN, GRU, LSTM, MLP, RF | diagnosis/ screening | K-fold CV, LOOCV | Cohens Kappa, Accuracy, Sensitivity, and Specificity |
| Kusmakar [20] | Classification | RF, SVM | diagnosis/ screening | LOOCV | Accuracy, sensitivity, specificity, |
| Kwon [21] | Classification | CNN | diagnosis/ screening | NR | Cohen’s kappa |
| Le [22] | Classification, regression | SVM | Prediction, diagnosis/ screening | K-fold CV | accuracy |
| McClure [23] | Classification | CNN | diagnosis/ screening | Training-test split | F1 score, accuracy, ROC |
| Papini [24] | Classification | CNN | diagnosis/ screening | Training-test split | Cohen’s kappa, Accuracy, sensitivity, specificity, PPV, PR AUC, ROC AUC |
| Park [25] | clustering | CAE | Prediction | NR | NR |
| Petrenko [26] | Classification | CNN | diagnosis/ screening, monitoring | Training-test split | accuracy |
| Rani [27] | Classification | kNN, SVM, NB, RF | diagnosis/ screening | K-fold CV | Accuracy, sensitivity, specificity, F1 score, AUC |
| Raschellà [28] | Classification | LDA, SVM, LR, NN, RF | diagnosis/ screening, | K-fold CV | Accuracy, sensitivity, specificity, |
| Rossi [29] | Classification | CNN | diagnosis/ screening | LOOCV, training-test split | Accuracy, sensitivity, specificity, |
| Ryser [30] | Classification | ABT,KNN | diagnosis/ screening | K-fold CV | Accuracy, sensitivity, specificity, PPV, NPV |
| Selvaraj [31] | Classification, regression | SVM | diagnosis/ screening, | LOOCV | Accuracy, sensitivity, specificity, |
| Shen [32] | Classification | CNN | diagnosis/ screening | K-fold CV, training-test split | Accuracy, sensitivity, specificity, F1 score, |
| Strumpf [33] | Classification | CNN | diagnosis/ screening | K-fold CV, training-test split | Accuracy, sensitivity, specificity, positive predictive value, negative predictive value, positive likelihood ratio, negative likelihood ratio, Kappa, Cohen’s Kappa coefficient |
| Tsouti [34] | Classification | NR | diagnosis/ screening, prediction | Training-test split | Accuracy, sensitivity, specificity |
| Van [35] | Classification | LSTM | diagnosis/ screening, prediction | K-fold CV | Accuracy, sensitivity, specificity, |
| Wang [36] | Classification | KNN, RF, SVM, XGBoost | diagnosis | NR | Accuracy, sensitivity, specificity |
| Wang [37] | Classification | CNN | diagnosis | K-fold CV, training-test split | Accuracy, sensitivity, specificity, F1 score, |
| Wu [38] | Classification | SVM | diagnosis/ screening | LOOCV | positive predictive value ,precision, and F1 score |
| Wu [39] | Classification, regression | DT, KNN, NB, RF | diagnosis/ screening | K-fold CV | Accuracy, sensitivity, specificity |
| Xu [40] | Classification, regression | NR | diagnosis/ screening | Training-test split | sensitivity, specificity, positive predictive value, negative predictive value, positive likelihood ratio, negative likelihood ratio, area under receiver operator characteristic curve area under the receiver operating characteristic (AUROC) curve and area under the precision-recall (AUPR) curve |
| Yeh [41] | classification | ANN | diagnosis | Training-test split | accuracy, sensitivity, specificity, positive predictive value, negative predictive value, positive likelihood ratio, negative likelihood ratio,, Cohen’s kappa coefficient, area under receiver operator characteristic curve area under the receiver operating characteristic (AUROC) curve and area under the precision-recall (AUPR) curve |
| Yeo [42] | Classification, regression | LDA, MLP, QDA, RF, SVM | diagnosis/ screening | Training-test split | sensitivity, specificity, positive predictive value, negative predictive value, accuracy, kappa coefficient, area under receiver operating characteristic score, area under precision-recall curve score |
| Yeo [43] | Classification, regression | CNN | diagnosis/ screening | Training-test split | sensitivity, specificity, positive predictive value, negative predictive value, accuracy, Cohen’s kappa coefficient, area under the receiver operating characteristic (AUROC) curve and area under the precision-recall (AUPR) curve |
| Yüzer [44] | Classification | ANN | diagnosis | Training-test split | NR |
| Zhang [45] | Classification | CNN | diagnosis/ screening | Training-test split | Accuracy |
| Zhou [46] | Classification | XGBoost | diagnosis/ screening | K-fold CV | Accuracy, sensitivity, specificity |

1. Benedetti, D., et al., *Obstructive Sleep Apnoea Syndrome Screening Through Wrist-Worn Smartbands: A Machine-Learning Approach.* Nat Sci Sleep, 2022. **14**: p. 941-956.

2. Chang, H.C., et al., *Portable Sleep Apnea Syndrome Screening and Event Detection Using Long Short-Term Memory Recurrent Neural Network.* Sensors (Basel), 2020. **20**(21).

3. Chen, M., et al., *Information-Based Similarity of Ordinal Pattern Sequences as a Novel Descriptor in Obstructive Sleep Apnea Screening Based on Wearable Photoplethysmography Bracelets.* Biosensors (Basel), 2022. **12**(12).

4. Chen, X., et al., *ApneaDetector: Detecting Sleep Apnea with Smartwatches.* Proc. ACM Interact. Mob. Wearable Ubiquitous Technol., 2021. **5**(2): p. Article 59.

5. Fallmann, S. and L. Chen. *Detecting Chronic Diseases from Sleep-Wake Behaviour and Clinical Features*. in *2018 5th International Conference on Systems and Informatics (ICSAI)*. 2018.

6. Fedorin, I. and K. Slyusarenko, *Consumer Smartwatches As a Portable PSG: LSTM Based Neural Networks for a Sleep-Related Physiological Parameters Estimation.* Annu Int Conf IEEE Eng Med Biol Soc, 2021. **2021**: p. 849-452.

7. Fedorin, I., K. Slyusarenko, and M. Nastenko, *Respiratory events screening using consumer smartwatches*. 2020. 25-28.

8. Ganglberger, W., et al., *Sleep apnea and respiratory anomaly detection from a wearable band and oxygen saturation.* Sleep Breath, 2022. **26**(3): p. 1033-1044.

9. Gu, W., et al., *Belun Ring Platform: a novel home sleep apnea testing system for assessment of obstructive sleep apnea.* J Clin Sleep Med, 2020. **16**(9): p. 1611-1617.

10. Hafezi, M., et al., *Sleep Apnea Severity Estimation From Tracheal Movements Using a Deep Learning Model.* IEEE Access, 2020. **8**: p. 22641-22649.

11. Hafezi, M., et al. *Sleep Apnea Severity Estimation from Respiratory Related Movements Using Deep Learning*. in *2019 41st Annual International Conference of the IEEE Engineering in Medicine and Biology Society (EMBC)*. 2019.

12. Hung, P.D., *Central Sleep Apnea Detection Using an Accelerometer*, in *Proceedings of the 1st International Conference on Control and Computer Vision*. 2018, Association for Computing Machinery: Singapore, Singapore. p. 106–111.

13. Jeon, S., Y.S. Lee, and S.H. Son, *Cascade Windows-Based Multi-Stream Convolutional Neural Networks Framework for Early Detecting In-Sleep Stroke Using Wristbands.* IEEE Access, 2023. **11**: p. 84944-84956.

14. Jeon, Y., K. Heo, and S.J. Kang, *Real-Time Sleep Apnea Diagnosis Method Using Wearable Device without External Sensors*. 2020. 1-5.

15. Ji, X., et al., *Airline Point-of-Care System on Seat Belt for Hybrid Physiological Signal Monitoring.* Micromachines, 2022. **13**(11): p. 1880.

16. Kanal, V., et al., *APSEN: Pre-screening Tool for Sleep Apnea in a Home Environment*. 2016.

17. Kim, W.P., et al., *Machine Learning-Based Prediction of Attention-Deficit/Hyperactivity Disorder and Sleep Problems With Wearable Data in Children.* JAMA Netw Open, 2023. **6**(3): p. e233502.

18. Kristiansen, S., et al., *Machine Learning for Sleep Apnea Detection with Unattended Sleep Monitoring at Home.* ACM Trans. Comput. Healthcare, 2021. **2**(2): p. Article 14.

19. Kristiansen, S., et al., *A clinical evaluation of a low-cost strain gauge respiration belt and machine learning to detect sleep apnea.* Smart Health, 2023. **27**: p. 100373.

20. Kusmakar, S., et al., *A machine learning model for multi-night actigraphic detection of chronic insomnia: Development and validation of a pre-screening tool.* Royal Society Open Science, 2021. **8**(6).

21. Kwon, S., et al., *At-home wireless sleep monitoring patches for the clinical assessment of sleep quality and sleep apnea.* Science Advances, 2023. **9**(21): p. eadg9671.

22. Le, T.Q., et al., *Wireless Wearable Multisensory Suite and Real-Time Prediction of Obstructive Sleep Apnea Episodes.* IEEE J Transl Eng Health Med, 2013. **1**: p. 2700109.

23. McClure, K., et al., *Classification and Detection of Breathing Patterns with Wearable Sensors and Deep Learning.* Sensors (Basel), 2020. **20**(22).

24. Papini, G.B., et al., *Wearable monitoring of sleep-disordered breathing: estimation of the apnea-hypopnea index using wrist-worn reflective photoplethysmography.* Sci Rep, 2020. **10**(1): p. 13512.

25. Park, S., et al., *Clustering Insomnia Patterns by Data From Wearable Devices: Algorithm Development and Validation Study.* JMIR Mhealth Uhealth, 2019. **7**(12): p. e14473.

26. Petrenko, A. *Breathmonitor: Sleep Apnea Mobile Detector*. in *2020 IEEE 2nd International Conference on System Analysis & Intelligent Computing (SAIC)*. 2020.

27. Rani, S., et al., *Differentiating acute from chronic insomnia with machine learning from actigraphy time series data.* Front Netw Physiol, 2022. **2**: p. 1036832.

28. Raschellà, F., et al., *Actigraphy Enables Home Screening of Rapid Eye Movement Behavior Disorder in Parkinson's Disease.* Ann Neurol, 2023. **93**(2): p. 317-329.

29. Rossi, M., et al., *SLEEP-SEE-THROUGH: Explainable Deep Learning for Sleep Event Detection and Quantification From Wearable Somnography.* IEEE J Biomed Health Inform, 2023. **27**(7): p. 3129-3140.

30. Ryser, F., et al., *Respiratory analysis during sleep using a chest-worn accelerometer: A machine learning approach.* Biomedical Signal Processing and Control, 2022. **78**: p. 104014.

31. Selvaraj, N. and R. Narasimhan, *Automated prediction of the apnea-hypopnea index using a wireless patch sensor.* 2014 36th Annual International Conference of the IEEE Engineering in Medicine and Biology Society, EMBC 2014, 2014. **2014**: p. 1897-900.

32. Shen, Q., et al., *Multitask Residual Shrinkage Convolutional Neural Network for Sleep Apnea Detection Based on Wearable Bracelet Photoplethysmography.* IEEE Internet of Things Journal, 2022. **9**(24): p. 25207-25222.

33. Strumpf, Z., et al., *Belun Ring (Belun Sleep System BLS-100): Deep learning-facilitated wearable enables obstructive sleep apnea detection, apnea severity categorization, and sleep stage classification in patients suspected of obstructive sleep apnea.* Sleep Health, 2023. **9**(4): p. 430-440.

34. Tsouti, V., et al., *Development of an automated system for obstructive sleep apnea treatment based on machine learning and breath effort monitoring.* Microelectronic Engineering, 2020. **231**: p. 111376.

35. Van Steenkiste, T., et al., *Portable Detection of Apnea and Hypopnea Events Using Bio-Impedance of the Chest and Deep Learning.* IEEE Journal of Biomedical and Health Informatics, 2020. **PP**: p. 1-1.

36. Wang, S., et al., *Machine Learning Assisted Wearable Wireless Device for Sleep Apnea Syndrome Diagnosis.* Biosensors, 2023. **13**(4): p. 483.

37. Wang, Z., et al., *Single-lead ECG based multiscale neural network for obstructive sleep apnea detection.* Internet of Things, 2022. **20**: p. 100613.

38. Wu, H.T., et al., *Phenotype-Based and Self-Learning Inter-Individual Sleep Apnea Screening With a Level IV-Like Monitoring System.* Front Physiol, 2018. **9**: p. 723.

39. Wu, S., et al., *Sleep apnea screening based on Photoplethysmography data from wearable bracelets using an information-based similarity approach.* Computer Methods and Programs in Biomedicine, 2021. **211**: p. 106442.

40. Xu, Y., et al., *Comparative study of a wearable intelligent sleep monitor and polysomnography monitor for the diagnosis of obstructive sleep apnea.* Sleep Breath, 2023. **27**(1): p. 205-212.

41. Yeh, E., et al., *Detection of obstructive sleep apnea using Belun Sleep Platform wearable with neural network-based algorithm and its combined use with STOP-Bang questionnaire.* PLoS One, 2021. **16**(10): p. e0258040.

42. Yeo, M., et al., *Respiratory Event Detection During Sleep Using Electrocardiogram and Respiratory Related Signals: Using Polysomnogram and Patch-Type Wearable Device Data.* IEEE J Biomed Health Inform, 2022. **26**(2): p. 550-560.

43. Yeo, M., et al., *Robust Method for Screening Sleep Apnea With Single-Lead ECG Using Deep Residual Network: Evaluation With Open Database and Patch-Type Wearable Device Data.* IEEE Journal of Biomedical and Health Informatics, 2022. **26**(11): p. 5428-5438.

44. Yüzer, A.H., et al., *A different sleep apnea classification system with neural network based on the acceleration signals.* Applied Acoustics, 2020. **163**: p. 107225.

45. Zhang, H., et al., *Long-Term Sleep Respiratory Monitoring by Dual-Channel Flexible Wearable System and Deep Learning-Aided Analysis.* IEEE Transactions on Instrumentation and Measurement, 2023. **72**: p. 1-9.

46. Zhou, G., et al., *Automatic monitoring of obstructive sleep apnea based on multi-modal signals by phone and smartwatch.* Annu Int Conf IEEE Eng Med Biol Soc, 2023. **2023**: p. 1-4.
